# Identification and characterization of *An-4*, a potential quantitative trait locus for awn development in rice

**DOI:** 10.1186/s12870-021-03055-w

**Published:** 2021-06-29

**Authors:** Baoxiang Qin, Taian Lu, Yibo Xu, Wei Shen, Fang Liu, Xuyang Xie, Yunzhen Li, Kejian Wang, Rongbai Li

**Affiliations:** 1 State Key Laboratory for Conservation and Utilization of Subtropical Agro-bioresources, Agricultural College, Guangxi University, Nanning, 530005 China; 2 State Key Laboratory of Rice Biology, China National Rice Research Institute, Chinese Academy of Agricultural Sciences, Hangzhou, 310006 China

**Keywords:** Rice (*Oryza sativa* L.), *An-4*, Awn development, Yield traits, Quantitative trait locus (QTL)

## Abstract

**Background:**

Awn of rice is an important domestication trait closely associated with yield traits. Therefore, the identification of genes for awn development is of great significance for the elucidation of molecular mechanism of awn development and the genetic improvement of yield traits in rice.

**Results:**

In this study, using chromosome segment substitution lines (CSSLs) derived from a long-awned Guangxi common wild rice (GXCWR, *Oryza rufipogon* Griff.) and a short-awned *indica* cultivar 9311, we identified *An-4*, a potential quantitative trait locus (QTL) for awn development. Then, *An-4* was fine mapped into a 56-kb region of chromosome 2, which contained four annotated genes. Among these four annotated genes, Os02g0594800 was concluded to be the potential candidate gene for *An-4*. *An-4* exhibited pleiotropic effects on awn development and several yield traits. Scanning electron microscopy (SEM) analysis showed that *An-4* significantly promoted awn development at Sp7 and Sp8 stage of spikelet development. Transcriptome analysis suggested that *An-4* might influence the development of awn by regulating the expression of genes related to growth, developmental process, channel regulation and extracellular region. By contrast to those of 9311, the expression level of *OsRR5* in CSSL128 was significantly down-regulated, whereas the expression levels of *OsCKX2* and *OsGA2ox5* in CSSL128 were significantly up-regulated. In addition, our study showed that *An-4* had additive effects with other genes for awn development, such as *An-1*, *An-2*/*LABA1* and *An-3*/*GAD1*/*RAE2*.

**Conclusions:**

The identification of *An-4* lays a foundation for cloning of *An-4* and further elucidation of the molecular mechanism of awn development. Moreover, the identification of favorable allelic variation of *An-4* from 9311 will be useful to improve rice yield traits.

**Supplementary Information:**

The online version contains supplementary material available at 10.1186/s12870-021-03055-w.

## Background

Rice is one of the most important grain crops and is responsible for feeding nearly a half of the world’s population [1]. Asian cultivated rice (*Oryza sativa* L.) is domesticated from common wild rice (*Oryza rufipogon* Griff.) [2, 3]. During domestication, numerous important traits, such as awn length, seed shattering, stem growth habit, and so on, have remarkably changed [4, 5]. The variations of these traits have increased the yield of rice. Therefore, isolation of the genes related to domestication traits and their favorable allelic variations are of great significance for the genetic improvement of rice yield traits.

Awn is a needle-like organ extending from the apex of lemma of spikelet. Awn of wild rice is beneficial to seed dissemination and protecting rice grains from animal predation [6, 7]. However, awn is unfavorable to seed storage and processing, so they were partially or completely eliminated by artificial selection during domestication from wild rice to cultivated rice.

Awn is a complicated trait regulated by many genes and generally exists in common wild rice [8–12]. Although several QTLs for awn development have been identified from common wild rice [13–15], only three of these QTLs, such as *An-1*, *An-2*/*LABA1*, and *An-3*/*GAD1*/*RAE2*, have been cloned and characterized thus far [16–21]. *An-1* encodes a bHLH transcription factor that positively regulates awn development and negatively regulates grain number in rice [16]. *An-2*/*LABA1* encodes a cytokinin synthesis enzyme that promotes awn elongation and decreases grain production in rice [17, 18]. *An-3*/*GAD1*/*RAE2* encodes a secreted peptide, the loss of *GAD1* function causes the increased grains number, shorter grains, and awnless phenotype [19–21].

In this study, we identified and characterized *An-4*, a potential QTL for awn development. *An-4* was narrowed down to a 56-kb region where Os02g0594800 was determined as the potential candidate gene for *An-4*. Our result showed that *An-4* had potential effects on some yield traits. Therefore, these results will not only help the future elucidation of molecular mechanism of awn development, but also facilitate the genetic improvement of rice yield traits.

## Results

### Molecular mapping of *An-4*

**Fig. 1:**
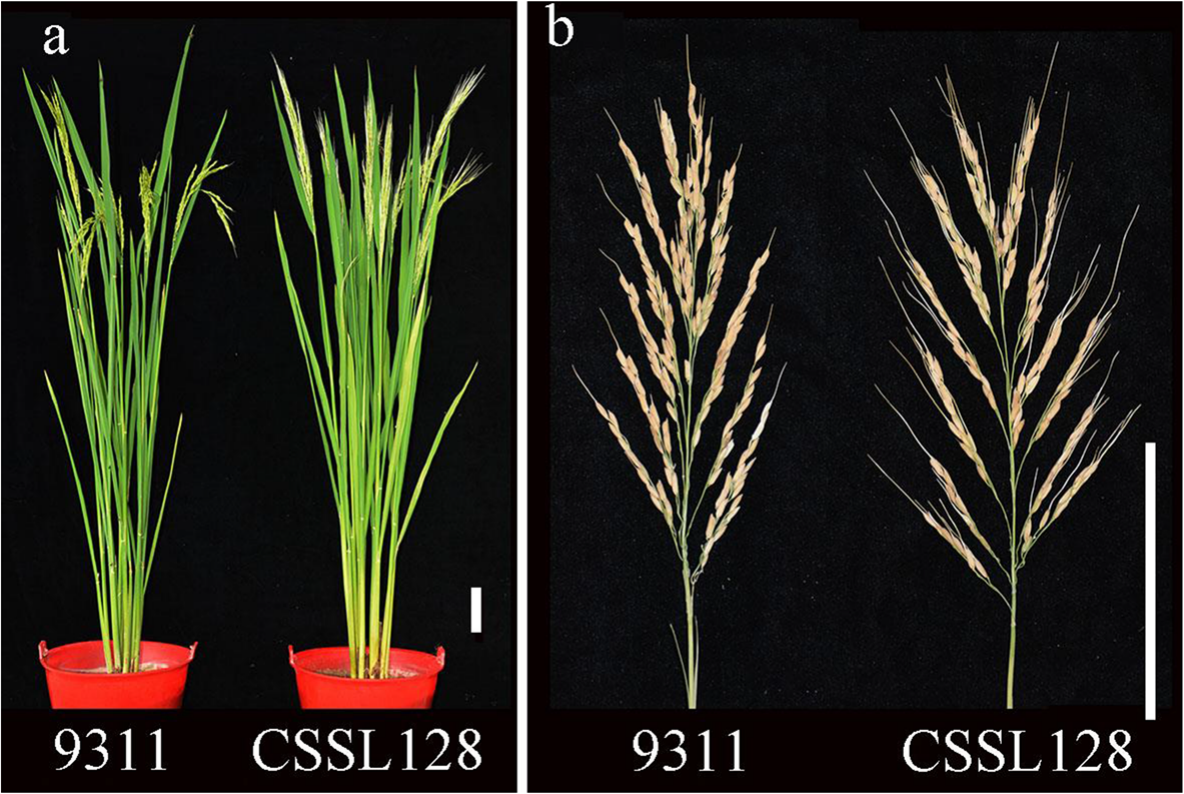
Phenotypic comparison between 9311 and CSSL128. **a** A comparison of plant architectures between 9311 and CSSL128. **b** A comparison of panicles between 9311 and CSSL128. *Scale bars* = 10 cm

**Fig. 2:**
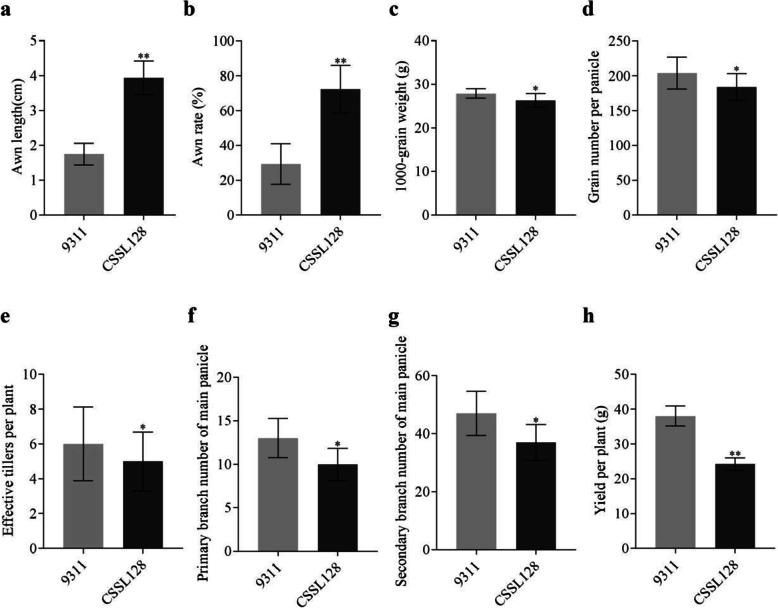
Comparison of awn length (**a**), awn rate (**b**), 1000-grain weight (**c**), grain number per panicle (**d**), effective tiller per plant (**e**), primary branch number of main panicle (**f**), secondary branch number of main panicle (**g**), yield per plant (**h**) between 9311 and CSSL128. *Significance at *p* < 0.05; **Significance at *p* < 0.01

To investigate the genetic basis of awn development in rice, we constructed a set of chromosome segment substitution lines (CSSLs) using the long-awned GXCWR and the short-awned *indica* variety 9311 as donor and recipient, respectively [19]. Of these lines, CSSL128 showed normal vegetative growth but a significant increase in the awn length and awn rate (Figs. 1a, b and 2a, b). The average length of awn and awn rate CSSL128 was 3.94 ± 0.34 cm and 72.32 ± 9.69%, respectively, whereas those of 9311 was 1.75 ± 0.22 cm and 29.31 ± 8.27%, respectively (Table 1).

**Table 1:** Comparison of yield related traits between 9311 and CSSL128

Yield related traits	9311	CSSL128
Awn length (cm)	1.75 ± 0.22	3.94 ± 0.34**
Awn rate (%)	29.31 ± 8.27	72.32 ± 9.69**
Plant height (cm)	114.10 ± 3.20	117.30 ± 2.30
Effective tillers per plant	6.00 ± 1.50	5.00 ± 1.20*
Length of main panicle (cm)	25.86 ± 1.70	24.88 ± 1.90
Primary branch number of main panicle	13.00 ± 1.60	10.00 ± 1.30*
Secondary branch number of main panicle	47.00 ± 5.40	37.00 ± 4.40*
Grain number per panicle	204.00 ± 16.20	184.00 ± 13.50*
Setting percentage (%)	87.12 ± 5.67	94.21 ± 4.78
1000-grain weight (g)	27.90 ± 0.75	26.31 ± 1.14*
Yield per plant (g)	38.05 ± 2.04	24.25 ± 1.26**
Grain length (mm)	9.20 ± 0.05	9.32 ± 0.08
Grain width (mm)	2.92 ± 0.03	2.86 ± 0.06
Length-width ratio	3.15 ± 0.04	3.26 ± 0.08
Grain circumference (mm)	24.01 ± 0.13	24.22 ± 0.21
Grain projected area (mm^2^)	19.42 ± 0.11	18.97 ± 0.10

Values are means ± SD (*n* = 20 plants)

*Significance at *p* < 0.05

**Significance at *p* < 0.01

To isolate the gene for long awn of CSSL128, we crossed CSSL128 with 9311 to construct a segregation population. We found that all the F_1_ individuals were long-awned. In the F_2_ population, the long-awned and short-awned individuals were segregated at an approximate rate of 3:1 (1121 long-awned: 350 short-awned; χ^2^ = 1.14; *P* > 0.05). These results show that the long awn trait of CSSL128 is controlled by a single dominant gene, named as *An-4* here.

**Fig. 3:**
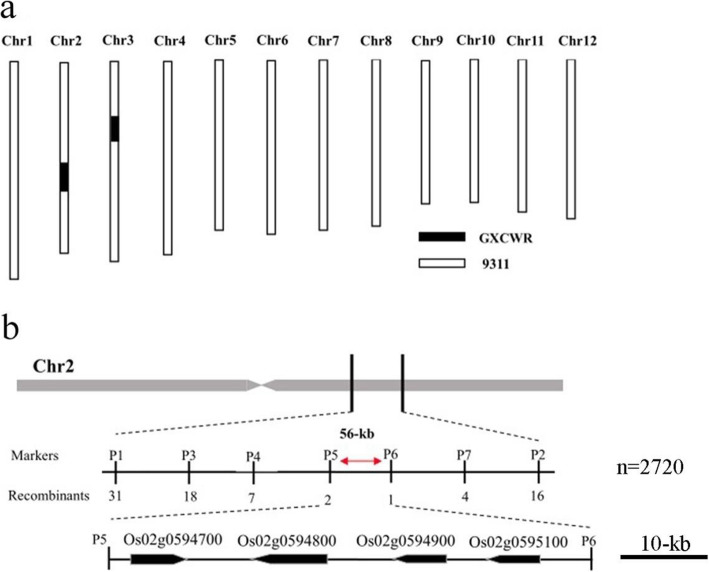
Molecular mapping of *An-4*. **a** Graphical genotype of chromosome segment substitution line CSSL128.White bar represents homozygous chromosomal fragment of 9311, black bar represents homozygous chromosomal fragment of GXCWR. **b** *An-4* was fine mapped into a 56-kb interval delimited by markers P5 and P6

To analyze the genetic background of CSSL128, 427 simple sequence repeat (SSR) and Insertion/Deletion (InDel) markers distributed on 12 chromosomes were initially selected for analysis of polymorphism between 9311 and GXCWR. Of these markers, 183 were polymorphic between the two parents and were used to further analyze the genetic variance between 9311 and CSSL128. Only five InDel markers located on chromosome 2 and 3 exhibited polymorphism between 9311 and CSSL128, which suggested that CSSL128 carries two chromosome segments on chromosome 2 and 3 from wild rice, respectively (Fig. 3a).

To primarily map *An-4*, 146 recessive individuals with short awn were used for genetic linkage analysis using above five markers on chromosome 2 and 3. *An-4* was primarily located between markers P1 and P2 positioned on chromosome 2. To fine-map *An-4*, a population of 2574 homozygous recessive individuals from a large F_3_ population was used to screen for recombinant individuals with markers P1 and P2, and 79 recombinant individuals were identified (Fig. 3b). And then, we further developed five polymorphic markers between markers P1 and P2, these five markers were used to survey 79 recombinant individuals. We found one recombinant individual for marker P6 and two recombinant individuals for marker P5. Therefore, *An-4* was fine mapped into a 56-kb interval delimited by markers P5 and P6 (Fig. 3b).

### Analysis of candidate genes in the 56-kb region

In the 56-kb candidate region, there were four annotated genes according to Rice Genome Annotation Project (http://rice.plantbiology.msu.edu/cgi-bin/gbrowse/rice/) (Fig. 3b). ORF1 (Os02g0594700) encoded a protein containing the BTBN3 family NPH3 domain, which mediated various blue light-induced responses, including phototropism, chloroplast movement, stomatal opening, and leaf flattening [22]. ORF2 (Os02g0594800) encoded a no apical meristem (NAM) family protein that was reported to regulate boundary formation, lateral organ separation and floral organ identity [23]. Mutation of *MtNAM* resulted in a reduced number of floral whorls and floral organs [23]. ORF3 (Os02g0594900) and ORF4 (Os02g0595100) encoded a glycosyl transferase family protein, respectively. Glycosyl transferase played key roles in maintaining plant normal growth and development, improving the abiotic stress tolerance of plants, regulating biosynthesis of plant secondary metabolites, enhancing the ability of plant disease resistance [24–27].

**Fig. 4:**
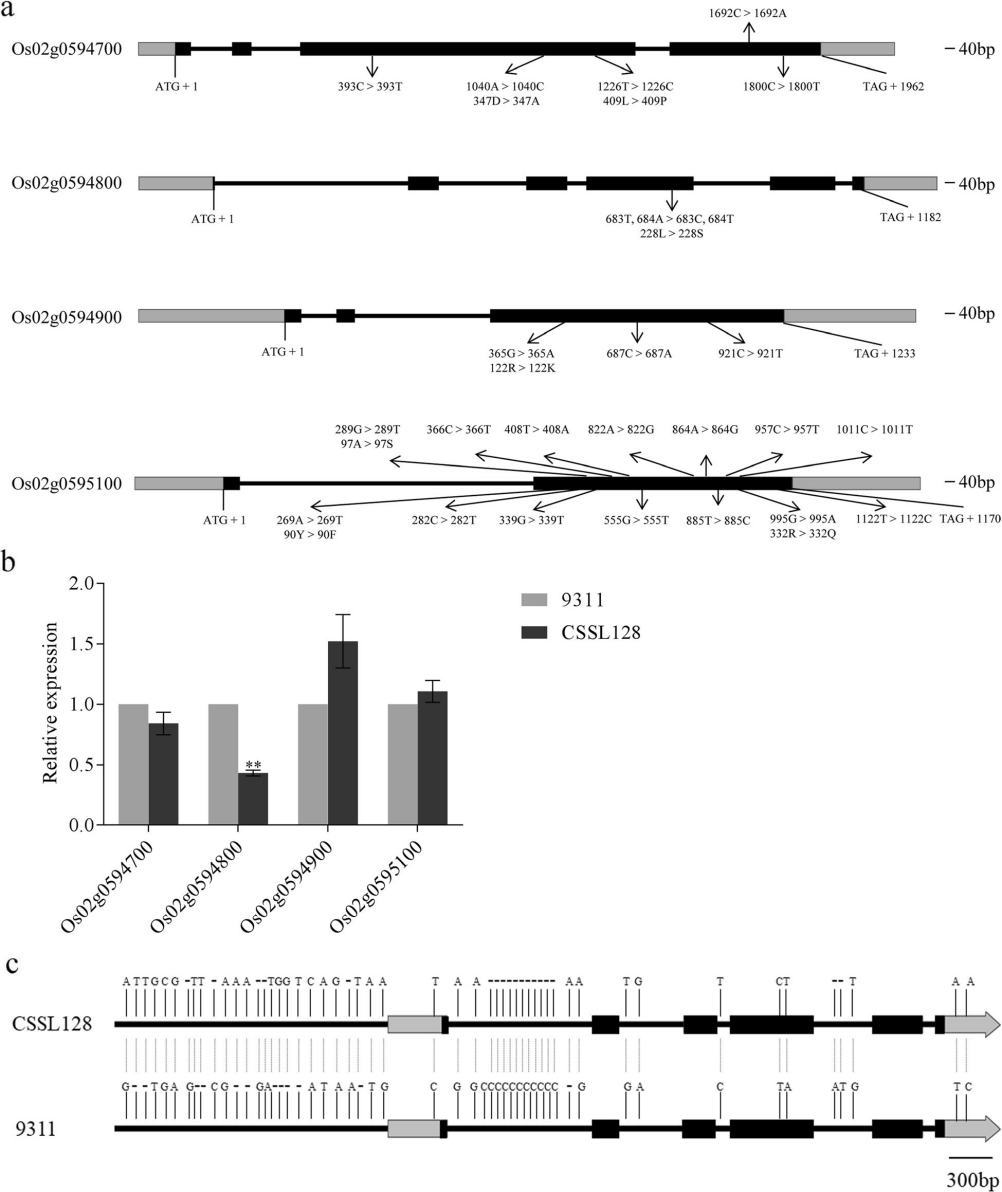
Analysis of four candidate genes. **a** Comparison of CDS and protein sequences of four candidate genes between 9311 and CSSL128. **b** Comparison of expression levels of four candidate genes between 9311 and CSSL128. **Significance at *p* < 0.01. **c** Comparison of the genomic sequence of Os02g0594800 between 9311 and CSSL128. The variations between 9311 and CSSL128 are indicated in this figure. Black bars represent 5′ upstream regions and introns, light-gray bars represent 5′ and 3′ untranslated regions, dark-gray bars represent coding regions, the short dashes represent single base pair deletions

We compared coding sequence (CDS) of these four candidate genes between 9311 and CSSL128 and found that all four candidate genes showed differences in CDS between 9311 and CSSL128 (Fig. 4a). For example, for Os02g0594700, there were five single nucleotide polymorphisms (SNPs), among these five SNPs, two caused amino acid changes, the A/C single-base substitution at position 1040 leaded to the change of Aspartic acid/Alanine, the T/C single-base substitution at position 1226 leaded to the change of Leucine/Proline; For Os02g0594800, there were two SNPs, the T/C and A/T single-base substitution at position 683 and 684 leaded to the change of Leucine/Serine; For Os02g0594900, there were three SNPs, among these three SNPs, only the G/A single-base substitution at position 365 resulted in the change of Arginine/Lysine; For Os02g0595100, there were fourteen SNPs, among these fourteen SNPs, three caused amino acid changes, the A/T single-base substitution at position 269 leaded to the change of Tyrosine/Phenylalanine, the G/T single-base substitution at position 289 leaded to the change of Alanine/Serine, the G/A single-base substitution at position 995 leaded to the change of Arginine/Glutarnine (Supplemental Table 1).

We also investigated the expression of all four annotated genes in young panicle by transcriptome analysis. The data demonstrated that compared with that of 9311, the expression of ORF2 (Os02g0594800) in CSSL128 was reduced by 1.63 times, whereas ORF1 (Os02g0594700), ORF3 (Os02g0594900) and ORF4 (Os02g0595100) showed no apparent different expression between 9311 and CSSL128 (Supplemental Table 2). Quantitative reverse transcription PCR (qRT-PCR) for these four annotated genes was also performed and the results of qRT-PCR were consistent with those of RNA sequencing analysis (Fig. 4b). These studies suggest that among four annotated genes, only Os02g0594800 showed differences both in coding region sequences and expression levels between 9311 and CSSL128. Therefore, genomic sequence of Os02g0594800 was further analyzed. We sequenced about 5.7-kb genomic sequence of CSSL128 and compared it with that of 9311. Eleven SNPs, eleven one-nucleotide indels, one two-nucleotide indels and one three--nucleotide indel were detected in the promoter region. Nine SNPs, one one-nucleotide indels, one two-nucleotide indels and one eleven-nucleotide indels were detected in the intron (Fig. 4c). These differences in the promoter region and intron of Os02g0594800 might result in different expression of Os02g0594800 between 9311 and CSSL128.

**Fig. 5:**
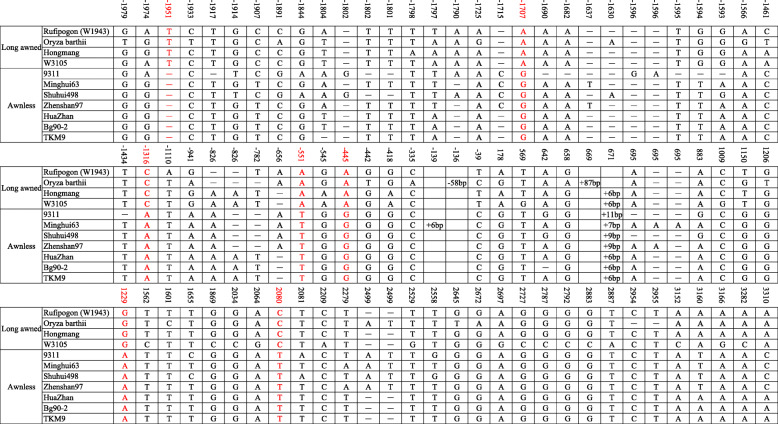
Comparative analysis of the genomic sequence of Os02g0594800 in 4 long-awned and 7 awnless accessions. The reference genome is Rufipogon (W1943), physical positions on Os02g0594800 are indicated across the top of the figure in base pairs, and the potential functional variations are indicated in red

To identify possible functional variations, 3 long-awned wild rice varieties and 1 long-awned cultivated variety, and 7 awnless cultivated rice varieties were randomly selected to sequence and compare the genomic sequence of Os02g0594800. We found that 90 loci showed variations in Os02g0594800 among 11 examined varieties. Among these variations, the variations at 7 loci might be the functional variation accounting for the awn differences (Fig. 5). For the variations at 7 loci, 5 were detected in the promoter region, for example, − 1951, − 1707, − 1316, − 551 and − 445 locus. 1 such as 1229 locus was detected in intron. 1 such as 2080 locus was detected in coding region, which was consistent with T/C substitution at 683 locus of CDS identified in our study.

### The potential effects of *An-4* on yield related traits

In addition to awn phenotype, CSSL128 also exhibited differences in several yield related traits (Table 1). For example, the 1000-grain weight, grain number per panicle and effective tiller per plant of CSSL128 were 94.30, 90.20 and 83.33% of those of 9311, respectively (Fig. 2c, d, e), while the primary branch number of main panicle and secondary branch number of main panicle were reduced to 76.92 and 78.72% of those of 9311 (Fig. 2f, g), respectively. The differences of these yield related traits caused yield per plant of CSSL128 reduced to 63.73% of that of 9311 (Fig. 2h). By comparison, no significant differences in plant height, length of main panicle, setting percentage, grain length, grain width, length-width ratio, grain circumference and grain projected area were detected between 9311 and CSSL128 (Supplemental Fig. 1).

### Comparative analysis of awn development between 9311 and CSSL128

**Fig. 6:**
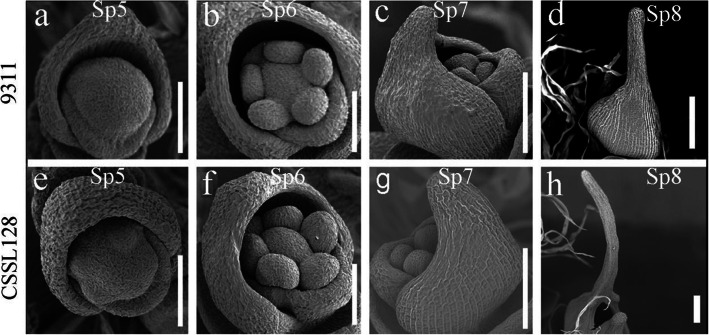
Scanning electron microscopy (SEM) images of spikelet at different developmental stage of 9311 and CSSL128. *Scale bars* = 100 μm

To determine the specific stage when awn differentiated between 9311 and CSSL128, we compared awn development between 9311 and CSSL128 using scanning electron microscopy (SEM). The rice spikelet development (Sp) stages were previously defined into 8 stages by Itoh et al. [28]. Lemma primordia was initiated at the Sp3 stage and then awn primordia extended from the apex of lemma primordial. We did not observe significant difference in awn development until the Sp6 stage between 9311 and CSSL128 (Fig. 6a, e, b, f). At the Sp7 stage, the awn primordia of CSSL128 extended much longer than that of 9311 (Fig. 6c, g). At the Sp8 stage when lemma and palea were gradually closed, the awn primordia of CSSL128 were significantly longer than that of 9311 (Fig. 6d, h). These results show that the awn primordia of CSSL128 grow faster than that of 9311, which cause CSSL128 to produce longer awn than 9311.

### Transcriptome analysis of 9311 and CSSL128 at the panicle differentiation stage

**Fig. 7:**
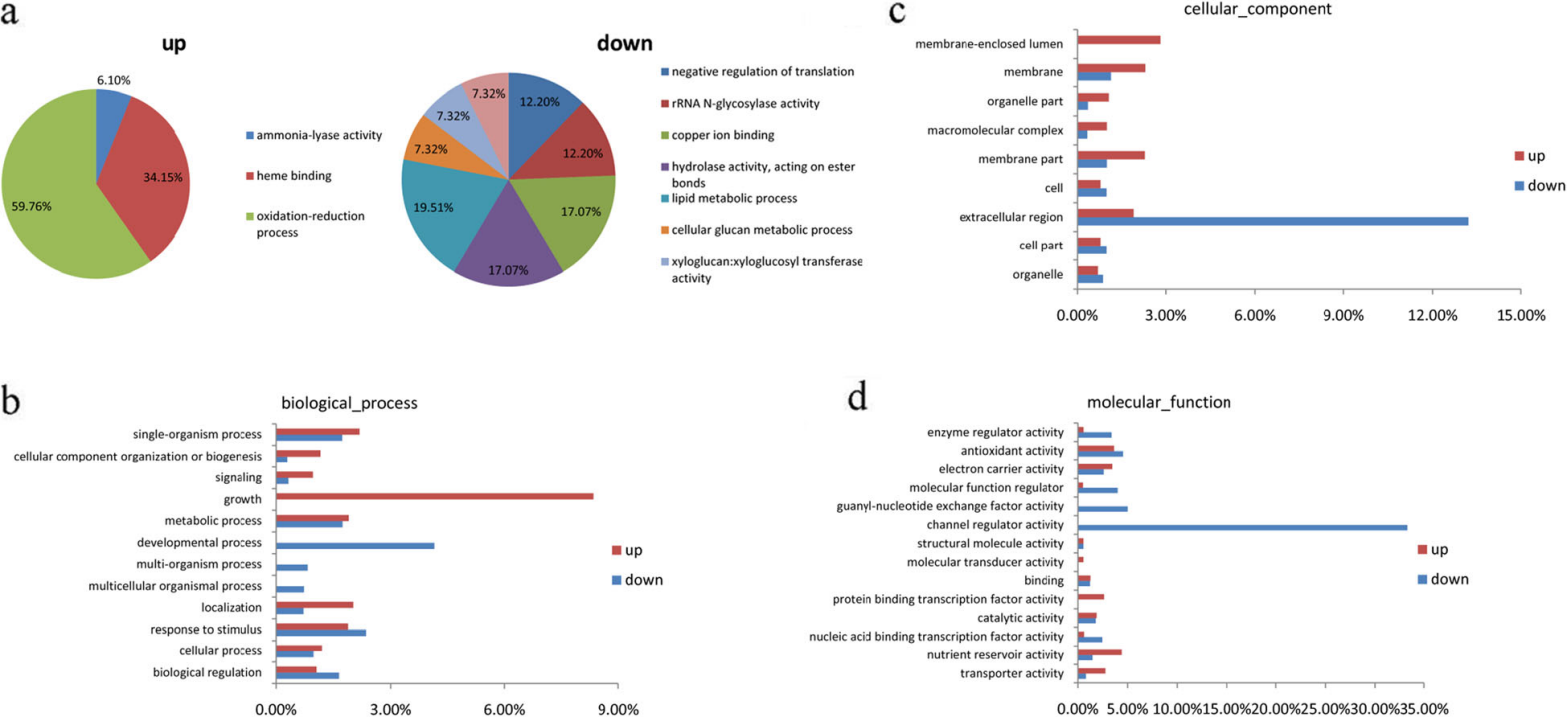
The proportion and GO classification of DEGs between 9311 and CSSL128

To investigate difference of gene expressions between 9311 and CSSL128 at the panicle differentiation stage, RNA sequencing was performed with young panicles. The data demonstrated that CSSL128 identified 1236 differential expressed genes compared with 9311, including 572 up-regulated and 664 down-regulated (Fig. 7a). Gene ontology (GO) analysis showed that all the differential genes were divided into three major categories: biological processes, cellular component and molecular function. Among three major categories, genes associated with growth were mostly enhanced (Fig. 7b), whereas genes involved to developmental process, channel regulator activity and extracellular region were significantly decreased (Fig. 7b, c, d). These results were consistent with previous report that the growth and development of plant organs required signaling pathways, these signaling pathways often connected several cellular components by channel regulator or protein translocation [29]. Therefore, *An-4* might influence the development of awn by regulating the expression of genes related to growth, developmental process, channel regulation and extracellular region.

### Comparison of expression level of the genes related to cytokinin, ethylene and gibberellin between 9311 and CSSL128

Cytokinin plays an important role in determining grain number and yield in rice. The cytokinin metabolism-related gene *OsCKX2* or *OsDST* was regarded to be negatively correlated with cytokinin concentration and grain number in rice, whereas the cytokinin-responsive genes *OsRRs* had been reported to be positively correlated with these two characters. To investigate the effect of *An-4* on expression of genes related to cytokinin metabolism and response, the expression levels of cytokinin metabolism-related genes *OsCKX2* and *OsDST* and cytokinin-responsive genes *OsRR1, OsRR2*, *OsRR3*, *OsRR4*, *OsRR5, OsRR6*, *OsRR7*, *OsRR8*, *OsRR9*, *OsRR10* and *OsRR11* were investigated by transcriptome analysis between 9311 and CSSL128. The data demonstrated that compared with those in 9311, among these genes, the expression level of *OsRR5* was significantly decreased and the expression level of *OsCKX2* was substantially increased, whereas the expression level of other genes were not significantly changed in CSSL128 (Supplemental Table 2).

In addition to cytokinin, ethylene and gibberellin (GA) function in plant development. To investigate the effect of *An-4* on expression of genes related to ethylene and GA, the expression levels of genes related to the metabolism and response of ethylene and GA were also investigated by transcriptome analysis. The data demonstrated that compared with those of 9311, the expression level of *OsGA2ox5* was significantly increased, whereas the expression levels of other genes related to the metabolism and response of ethylene and GA were not significantly changed in CSSL128 (Supplemental Table 2).

**Fig. 8:**
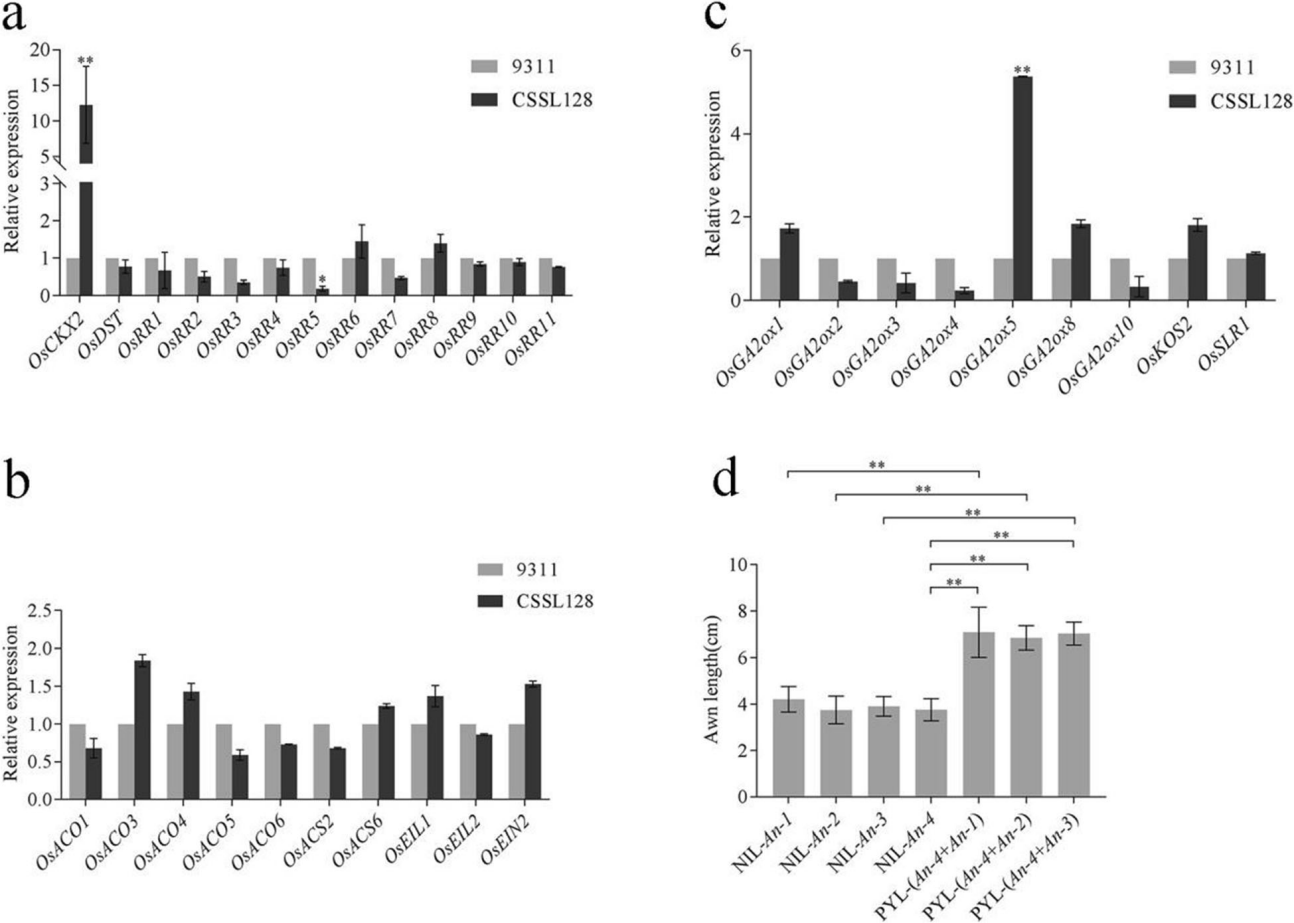
The effect analysis of *An-4* on genes related to cytokinin, ethylene, GA, *An-1*, *An-2* and *An-3*. **a** Comparison of expression level of the genes related to cytokinin metabolism and response between 9311 and CSSL128. **b** Comparison of expression level of the genes related to ethylene metabolism and response between 9311 and CSSL128. **c** Comparison of expression level of the genes related to GA metabolism and response between 9311 and CSSL128. **d** Analysis of pyramiding effect of *An-4* and *An-1*, *An-2*, *An-3*. *Significance at *p* < 0.05; **Significance at *p* < 0.01

To validate the reliability of RNA sequencing data, qRT-PCR for above genes were performed. The results of qRT-PCR were consistent with those of RNA sequencing analysis (Fig. 8a, b, c), suggesting that RNA sequencing data was reliable.

### *An-4* has additive effects with *An-1*, *An-2*/*LABA1* and *An-3*/*GAD1*/*RAE2*

The roles of *An-1*, *An-2*/*LABA1* and *An-3*/*GAD1*/*RAE2* in awn development had been previously determined. To analyze the relationships between *An-4* and these three genes, respectively, we developed nearly isogenic line (NIL) for these four genes and a set of pyramiding lines (PYLs) by marker-assisted selection (MAS), and then analyzed the independent and combined effects of *An-4* and these three genes in awn development.

The awn length of NIL-*An-1*, NIL-*An-2*, NIL-*An-3* and NIL-*An-4* was 4.21 ± 0.39 cm, 3.75 ± 0.42 cm, 3.91 ± 0.30 cm, and 3.76 ± 0.34 cm, respectively, whereas the awn length of pyramiding line PYL-(*An-4* + *An-1*), PYL-(*An-4* + *An-2*) and PYL-(*An-4* + *An-3*) was 7.24 ± 0.61 cm, 6.85 ± 0.37 cm and 7.03 ± 0.35 cm, respectively. Significant difference analysis showed that PYL-(*An-4* + *An-1*) significantly exhibited longer awn than *An-4* and *An-1*. PYL-(*An-4* + *An-2*) significantly exhibited longer awn than *An-4* and *An-2*. Similarly, PYL-(*An-4* + *An-3*) significantly exhibited longer awn than *An-4* and *An-3* (Fig. 8d). These results clearly suggest that *An-4* has an additive effect with *An-1*, *An-2*/*LABA1* and *An-3*/*GAD1*/*RAE2*.

## Discussion

Rice domestication traits include seed shattering, seed dormancy, awn, plant architecture, hull color and so on. Among them, awn is unfavorable to seed storage and processing, most cultivated rice bear no awns or very short awns. However, the causal genetic factors responsible for the loss of awn in cultivated rice remain largely unknown. Therefore, exploration of new genes for awn development would contribute to understanding the molecular mechanisms of rice domestication.

In this study, we identified and characterized *An-4*, a potential QTL for awn development. To isolate *An-4*, a map-based cloning strategy was employed and *An-4* was finally narrowed down within a 56-kb region on the long arm of chromosome 2. Around *An-4* locus, *qAWNL2* had been reported to be associated with awn development [30]. However, the physical distance between *An-4* and *qAWNL2* was about 3.8-Mb. Therefore, *An-4* and *qAWNL2* cannot be the same gene. In this region, there were four annotated genes. Among them, no known gene was reported, so *An-4* was considered as a novel gene for awn development in rice. Among these four annotated genes, only Os02g0594800 showed differences both in genomic sequences and expression levels between 9311 and CSSL128. Moreover, the variations identified from 7 different loci of Os02g0594800 might be the functional variations accounting for the awn differences. In additional, Os02g0594800 encoded a NAM family protein, which had been reported to affect organs morphogenesis in plant, especially floral organ. Its function might be most closely associated with phenotype of awn development. By comparison, the function of rest three genes in organs morphogenesis, especially floral organ, has not yet reported. Therefore, we think that Os02g0594800 is most possible candidate gene regulating awn development in CSSL128, more work such as complementary and knockout test are needed to determine which gene is the candidate gene for the *An-4*.

Compared with 9311, CSSL128 exhibited differences in several yield related traits. For example, effective tillers per plant, primary branch number of main panicle, secondary branch number of main panicle, grain number per panicle, 1000-grain weight and yield per plant were significantly decreased in CSSL128, which suggests that *An-4* may be a pleiotropic gene. Previous reports had suggested that the genes responsible for awn development had pleiotropic effects on several yield related traits, for example, *An-1* and *GAD1* decreased grain number per panicle and yield per plant [16, 20]. *An-2* decreased grain number per panicle and tiller number per plant [17]. *An-3,* an allele of *GAD1*, was showed to negatively regulated 1000-grain weight, grain length, and length-width ratio [19]. Moreover, analysis for *An-1, An-2* and *GAD1* showed that the genetic variation of these three genes caused awn loss and increase grain number and yield in cultivated rice. These findings suggest that long awn reduced yield per plant in rice and subsequently was under strong artificial selection during domestication. However, the differences of yield related traits between 9311 and CSSL128 might be resulted from the other genes because of two different segments from wild rice in CSSL128, or this might be caused by the linkage drag. In view of little interference of genetic background, the NIL, complementary or knockout line will be ideal material for improving the precise of phenotypic evaluation of *An-4*. Therefore, it will be needed to develop NIL, complementary or knockout line to analyze the effect of *An-4* on yield related traits. Rice breeding has mostly depended on genetic variations available among different species, the potential effects of genes responsible for awn development on yield related traits suggests that it might be an efficient strategy for genetic improvement of yield related traits to explore favorable allelic variations of genes responsible for awn development and transfer them to cultivars varieties.

The functions of cytokinin, ethylene and GA in plant development had been illustrated in a number of reports. In our study, the effects of *An-4* on expression of genes related to cytokinin, ethylene and GA were examined. We found that compared with those of 9311, the expression levels of *OsCKX2* and *OsGA2ox5* were significantly increased, whereas the expression level of *OsRR5* was substantially decreased in CSSL128. The result suggests that *An-4* might regulate the awn and yield traits by the interaction of cytokinin and GA. It is well known that cytokinin and GA function in plant development as a key modulator of cell expansion and elongation. Therefore, we suggest that at the apex of lemma, *An-4* might promote continuous cell division and induce awn primordia formation, whereas in the early stage of inflorescence formation, *An-4* might inhibit cell division, decrease meristematic activity and subsequently decrease branch number, grain number per panicle and yield per plant.

So far, three genes for awn development, namely *An-1*, *An-2*/*LABA1* and *An-3*/*GAD1*/*RAE2*, have been cloned and characterized. In this study, we found that *An-4* had an additive effect with *An-1*, *An-2*/*LABA1* and *An-3*/*GAD1*/*RAE2*, which was consistent with previous study that *GAD1* might have an additive effect with *An-1* and *An-2*/*LABA1*. These results suggest that awn is a complicated trait regulated by many genes and the pyramiding of these genes confers long awn phenotype of wild rice.

## Conclusions

In this study, we identified *An-4*, a potential QTL for awn development. The *indica* variety 9311 allele of *An-4* could increase yield per plant, so it could be useful for improving rice yield trait. *An-4* was fine mapped within a 56-kb region where Os02g0594800 was determined as the most probable candidate gene for *An-4*. Complementary and knockout test will be carried out in the future to validate the function of the candidate gene. To understand how *An-4* affects awn development and yield traits, further study is needed to clarify their molecular and biological functions.

## Materials and methods

### Plant materials

In this study, plant material of *Oryza rufipogon* Griff. GXCWR and *indica* variety 9311 were acquired from Agricultural College, Guangxi University (ACGU), China (22.84^。^N, 108.48^。^E). Identification of the plant materials were made by the ACGU and original plants were acquired from Guangxi Academy of Agricultural Sciences (www.gxaas.net). The voucher specimens were deposited at rice germplasm resource nursery of ACGU.

Chromosome segment substitution line CSSL128 was constructed using the long-awned Guangxi common wild rice (GXCWR, *Oryza rufipogon* Griff.) and the short-awned *indica* variety 9311 as donor and recipient, respectively. CSSL128 was crossed with the genetic background parent 9311 to generate F_1_ and a F_1_ individual plant was self-crossed to generate the F_2_ population. A total of 146 short-awn individuals were used for primarily mapping of *An-4*. Then, using the closely linked molecular markers, the heterozygous plants in the F_2_ population were selected for self-crossed to generate F_3_ population. A total of 2574 short-awn individuals from F_3_ population were used for fine mapping of *An-4*.

NILs of *An-1*, *An-2*/*LABA1*, *An-3*/*GAD1*/*RAE2* and *An*-*4* were developed with 9311 as a receptor. We backcross 9311 with CSSL95 (harboring *An-1*), CSSL5 (harboring *An-2*/*LABA1*), CSSL138 (harboring *An-3*/*GAD1*/*RAE2*) and CSSL128 (harboring *An-4*) for four generations, respectively, and then self-crossed to produce BC_4_F_2_ populations. Through MAS, NIL-*An-1*, NIL-*An-2*, NIL-*An-3* and NIL-*An-4* were developed from BC_4_F_2_ populations. The genetic backgrounds of these four NILs were analyzed by 183 polymorphic markers between 9311 and GXCWR, and it was found that these four NILs showed 95.21, 96.37, 97.41 and 98.17% genetic identity to 9311, respectively. These data suggests that NIL-*An-1*, NIL-*An-2*, NIL-*An-3* and NIL-*An-4* had been successfully constructed.

PYLs of *An-4* and other three genes were constructed by crossing *An-4* and other three genes such as *An-1*, *An-2*/*LABA1* and *An-3*/*GAD1*/*RAE2*, respectively. NIL-*An-4* was crossed with NIL-*An-1*, NIL-*An-2*, NIL-*An-3*, respectively. Three pyramided lines such as PYL-(*An-4* + *An-1*), PYL-(*An-4* + *An-2*) and PYL-(*An-4* + *An-3*) were selected from F_2_ populations through MAS. The genetic backgrounds of three PYLs were examined by 183 polymorphic markers between 9311 and GXCWR, and we found that these PYLs almost had same genetic background as 9311 except for the homozygous *An-4* locus and other awn gene locus, such as *An-1*, *An-2*/*LABA1* and *An-3*/*GAD1*/*RAE2*. These data suggest that PYL-(*An-4* + *An-1*), PYL-(*An-4* + *An-2*) and PYL-(*An-4* + *An-3*) had been successfully developed.

All the plants were grown in the experimental field of Agricultural College, Guangxi University, Nanning, China and were grown under normal growth conditions. The traits of all plants were investigated at maturity.

### Gene annotation and sequencing

The Rice Genome Annotation Project (http://rice.plantbiology.msu.edu/cgi-bin/gbrowse/rice/) was used for gene annotation. Four candidate genes were amplified and sequenced using CSSL128 DNA as a template. Then, NCBI Blast was used to compare the CDS and protein sequence of four candidate genes between 9311 and CSSL128.

### Phenotypic evaluation

20 plants were randomly selected from 9311 and CSSL128 for phenotypic evaluation at the maturity stage, respectively. The awn length was considered to be the distance from the root of outer glume to the end of awn. The awn length of the top of all branches of each plant was measured and the average value represented the average awn length of the plant. The awn rate per panicle was indicated by the formulas: the number of awned seed / total number of seed per panicle × 100%. The average statistical method of the awn rate was same as that of the awn length. The tiller with more than 5 grains was considered as effective tiller. Primary branches number of main panicle, secondary branches number of main panicle, grain number per panicle and setting percentage were manually counted. After all seeds of each plant were dried, threshed and removed awn, 1000-grain weight, yield per plant, grain length, grain width, grain length-width ratio, grain circumference, grain projected area were measured by scanner and counted. The plant height was indicated by the vertical distance from the bottom of rice to the top of flag leaf. The length of main panicle was indicated by the length from the stem node to the top of main panicle. The average value of these traits of 20 plants was used to represent the average value of the corresponding material. Finally, the statistical phenotypic data was analyzed using *T*-Test for significant difference.

### Scanning electron microscopy

In order to observe the development of rice spikelet, we took the spikelet at different developmental stage, putted them in 2.5% glutaraldehyde fixative solution and fixed them at 4 °C for more than 12 h, then dehydrated through an ethanol series and used them before observation carbon dioxide critical point dryer for drying. The dried spikes were plated with gold and observed at 15 kV using Hitachi S-2460 SEM.

### Transcriptome sequencing

Total RNA was extracted from young panicle at panicle differentiation stage using TRIzol reagent (Invitrogen, USA) and RNeasy Mini Kit (Qiagen, GER). Three biological replicates were performed for each sample. The quality and concentration of RNA were measured by Agilent 2100 bioanalyzer (Agilent Technologies, Palo Alto, CA, USA), Nano Drop (Thermo Fisher Scientific, USA) and 1% agrose gel electrophoresis. And then, cDNA libraries were constructed with NEBNext® UltraTM RNA library Prep Kit for Illuminae®(*BioLabs*, USA). The library was used for RNA-seq with an Illumina Hi-Seq sequencer (Illumina, San Diego, CA, USA) and 150 bp paired-end reads were generated. The average raw reads of 9311 and CSSL128 were 2,177,319 and 2,490,634, respectively. Raw read datasets were quality checked and filtered by SolexaQA and read by FastQC (bioinformatics.babraham.ac.uk/projects/fastqc/). The TopHat2 software was used to clean up the data and aligned to the reference genome. We used (RPKM) per million reads per thousand bases to determine the gene expression level. HTSeq (htseq.readthedocs) was used to calculate the gene count and DESeq (huber.embl.de/Users/Anders/DESeq/) was used as the input of differential gene expression analysis. Finally, the differentially expressed genes (DEGs) were screened according to the results of the difference multiple and *P*-value significance test. DEGs were defined by fold change values of normalized FPKM (log2(FC), FC designates fold change) among pair-wise sample groups and *P*-values were adjusted using the Benjamini and Hochbrg method. Then the GO and KEGG annotations were analyzed by DEG gene set enrichment analysis. The analysis was performed using a custom-written R script (https:// github.com/IdoBar/Trinotate_GSEA_plotteR).

### Real-time quantitative RT-PCR

Total RNA was extracted using Fast Pure Plant Total RNA Isolation Kit (Vazyme, CHN) and was reverse transcribed with HiScript III RT SuperMix for the qPCR Kit (Vazyme, CHN). The qRT-PCR was performed on a qTOWER3 real-time system (analytikjena) using diluted cDNA. 5′ and 3′ rapid amplification of cDNA ends were performed with the ChamQ Universal SYBR qPCR Master Mix (Vazyme, CHN) following the manufacturer’s instructions. Rice gene *UBI* was used as the control to normalize all data. Each experiment was repeated 3 times, and the relative quantitative method 2^-△△CT^ (DDCT) was used to evaluate the quantitative change.

### Primers

43 primers used in this study were listed in Supplemental Table 3. The primers for P1-P7 were developed based on the diversity between the genomic DNA sequence of *Oryza rufipogon* and 9311 in the region spanning *An-4* locus. The sequences of primers were designed using the DNASTAR-Lasergene v6 software.

## Supplementary Information

**Additional file 1: Supplemental Table 1.** Comparative analysis of CDS of four candidate genes between 9311 and CSSL128. **Supplemental Table 2.** Transcriptome data of genes related to cytokinin, ethylene, GA and four annotated genes between 9311 and CSSL128. **Supplemental Table 3.** List of primers for molecular mapping and qRT-PCR. **Supplemental Fig. 1.** Comparison of some yield related traits between 9311 and CSSL128.

## Data Availability

The data sets used and/or analysed during the current study are available from the corresponding author on reasonable request.

## Abbreviations

GXCWR: Guangxi common wild rice
QTL: Quantitative trait locus
SEM: Scanning electron microscopy
Sp: Spikelet development
CSSLs: Chromosome segment substitution lines
SNPs: Single nucleotide polymorphisms
SSR: Simple sequence repeat
InDel: Insertion/Deletion
qRT-PCR: quantitative reverse transcription PCR
CDS: Coding sequence
GO: Gene ontology
GA: Gibberellin
