# Supplementary material for: Identification and characterization of An-4, a potential quantitative trait locus for awn development in rice
Source: BMC Plant Biol. 2021 Jun 29;21:298. doi: 10.1186/s12870-021-03055-w (PMC8240324; doi:10.1186/s12870-021-03055-w)
Supplement: Supplementary file 1 — Additional file 1: Supplemental Table 1. Comparative analysis of CDS of four candidate genes between 9311 and CSSL128. Supplemental Table 2. Transcriptome data of genes related to cytokinin, ethylene, GA and four annotated genes between 9311 and CSSL128. Supplemental Table 3. List of primers for molecular mapping and qRT-PCR. Supplemental Fig. 1. Comparison of some yield related traits between 9311 and CSSL128. [file 12870_2021_3055_MOESM1_ESM.docx]

**Supplemental Table. 1 Comparative analysis of CDS of four candidate genes between 9311 and CSSL128.**

**Os02g0594700**

| CSSL128 | 1 ATGAAGTTTATGAAACTTGGCACCCGGCCTGACACCTTCT 40 |
| --- | --- |
| 9311 | 1 ATGAAGTTTATGAAACTTGGCACCCGGCCTGACACCTTCT 40 |
|  |  |
| CSSL128 | 41 ACTCCAACGAATCCGTGAGGTCTGTCTGCACAGAGGTCGC 80 |
| 9311 | 41 ACTCCAACGAATCCGTGAGGTCTGTCTGCACAGAGGTCGC 80 |
|  |  |
| CSSL128 | 81 CACTGACCTGCAAATCTTGGTGGGTGACTGCTTGTATCAG 120 |
| 9311 | 81 CACTGACCTGCAAATCTTGGTGGGTGACTGCTTGTATCAG 120 |
|  |  |
| CSSL128 | 121 CTTCACAAGTTTCCTCTGCTGTCGAAATGCCTGCTTCTGC 160 |
| 9311 | 121 CTTCACAAGTTTCCTCTGCTGTCGAAATGCCTGCTTCTGC 160 |
|  |  |
| CSSL128 | 161 AAGCGCTGTGCGCCGAGTCCGGATGCGGCGGCAATGGCGG 200 |
| 9311 | 161 AAGCGCTGTGCGCCGAGTCCGGATGCGGCGGCAATGGCGG 200 |
|  |  |
| CSSL128 | 201 CGACGTGATCGAGCTCCCGGGGTTCCCGGGCGGCGTCGAG 240 |
| 9311 | 201 CGACGTGATCGAGCTCCCGGGGTTCCCGGGCGGCGTCGAG 240 |
|  |  |
| CSSL128 | 241 GCGTTCGACGCGTGCGCCAAGTTCTGCTACGGCATCACCG 280 |
| 9311 | 241 GCGTTCGACGCGTGCGCCAAGTTCTGCTACGGCATCACCG 280 |
|  |  |
| CSSL128 | 281 TCACGGTGAGCGCGCGGAACCTCGTCCCGCTCCGCTGCGC 320 |
| 9311 | 281 TCACGGTGAGCGCGCGGAACCTCGTCCCGCTCCGCTGCGC 320 |
|  |  |
| CSSL128 | 321 CGCGGCGCACCTCGGCATGTCGGAGGCCGCCGACCGCGGC 360 |
| 9311 | 321 CGCGGCGCACCTCGGCATGTCGGAGGCCGCCGACCGCGGC 360 |
|  |  |
| CSSL128 | 361 AACCTCGCCGCCAAGCTCGACGCGTTCCTCGCTTCCTGCC 400 |
| 9311 | 361 AACCTCGCCGCCAAGCTCGACGCGTTCCTCGCCTCCTGCC 400 |

| CSSL128 | 401 TCCTCCGGCGATGGAAGGACGCGCTCGCCGTGCTCAACTC 440 |
| --- | --- |
| 9311 | 401 TCCTCCGGCGATGGAAGGACGCGCTCGCCGTGCTCAACTC 440 |
|  |  |
| CSSL128 | 441 GACGCGCCACTGCGCGCCGCTCTGCGAGGACATCGGCCTC 480 |
| 9311 | 441 GACGCGCCACTGCGCGCCGCTCTGCGAGGACATCGGCCTC 480 |
|  |  |
| CSSL128 | 481 ACCTCCCGGTGCGTGGACGCCGTCGCGGCGCTCATCGCGA 520 |
| 9311 | 481 ACCTCCCGGTGCGTGGACGCCGTCGCGGCGCTCATCGCGA 520 |
|  |  |
| CSSL128 | 521 GCCCCGCCGCCCTCCCGGCGCACTCCTCGTCGGCGTCGCC 560 |
| 9311 | 521 GCCCCGCCGCCCTCCCGGCGCACTCCTCGTCGGCGTCGCC 560 |
|  |  |
|  |  |
| CSSL128 | 561 GTGGTGGGCGCACGACGTCGCCGAGCTCGGGGTTGACCTG 600 |
| 9311 | 561 GTGGTGGGCGCACGACGTCGCCGAGCTCGGGGTTGACCTG 600 |

| CSSL128 | 601 TTCTGGCGCATCATGGTGGCCGTCAAGGCCACCGGCGCCG 640 |
| --- | --- |
| 9311 | 601 TTCTGGCGCATCATGGTGGCCGTCAAGGCCACCGGCGCCG 640 |
|  |  |
| CSSL128 | 641 TCCACGAGAAGACCGTCGGCGACGCGCTCAAGGCGTACGC 680 |
| 9311 | 641 TCCACGAGAAGACCGTCGGCGACGCGCTCAAGGCGTACGC 680 |
|  |  |
| CSSL128 | 681 GCGCCGGTGGCTGCCCAACGTCGCCAAGGACGGGATCGTC 720 |
| 9311 | 681 GCGCCGGTGGCTGCCCAACGTCGCCAAGGACGGGATCGTC 720 |
|  |  |
| CSSL128 | 721 GTCGGCGCCGACCAGCCGTTCGACGGGGCCGGCAATGGCG 760 |
| 9311 | 721 GTCGGCGCCGACCAGCCGTTCGACGGGGCCGGCAATGGCG 760 |
|  |  |
| CSSL128 | 761 GCGACGGCGGCAATGCCAGCGTCAAGCAGATCGCCACGAG 800 |
| 9311 | 761 GCGACGGCGGCAATGCCAGCGTCAAGCAGATCGCCACGAG 800 |

| CSSL128 | 801 GCACCGCCTCCTCCTCGAGAAGATCGTGAGCCTGATCCCG 840 |
| --- | --- |
| 9311 | 801 GCACCGCCTCCTCCTCGAGAAGATCGTGAGCCTGATCCCG 840 |
|  |  |
| CSSL128 | 841 GCGGAGAGGGACGCCGTCTCCTGCAGCTTCCTCCTCAAGC 880 |
| 9311 | 841 GCGGAGAGGGACGCCGTCTCCTGCAGCTTCCTCCTCAAGC 880 |
|  |  |
| CSSL128 | 881 TCCTCAAGGCGGCGAACATCCTCAGCGCGTCCGCCACGTC 920 |
| 9311 | 881 TCCTCAAGGCGGCGAACATCCTCAGCGCGTCCGCCACGTC 920 |
|  |  |
| CSSL128 | 921 CAGGGCGGAGCTGGTGCGGAGGGTGGCGTGGCAGCTCGAG 960 |
| 9311 | 921 CAGGGCGGAGCTGGTGCGGAGGGTGGCGTGGCAGCTCGAG 960 |
|  |  |
| CSSL128 | 961 GAGGCCACCGTCGGCGACCTCCTGATCCCGTCGCTGTCGT 1000 |
| 9311 | 961 GAGGCCACCGTCGGCGACCTCCTGATCCCGTCGCTGTCGT 1000 |
|  |  |
| CSSL128 | 1001 GCGTGTCCGAGACGCTGTACGACGTGGACGCCGTGGCGGC 1040 |
| 9311 | 1001 GCGTGTCCGAGACGCTGTACGACGTGGACGCCGTGGCGGA 1040 |
|  |  |
| CSSL128 | 1041 CATCCTCGACGAGTTCGCCCTGCGCCACGCCGCCGCGCCG 1080 |
| 9311 | 1041 CATCCTCGACGAGTTCGCCCTGCGCCACGCCGCCGCGCCG 1080 |
|  |  |
| CSSL128 | 1081 CCGCCGCCGGTGGCGCTGGCAGTGAGCCCCGACGACGACG 1120 |
| 9311 | 1081 CCGCCGCCGGTGGCGCTGGCAGTGAGCCCCGACGACGACG 1120 |
|  |  |
| CSSL128 | 1121 ACGACAGCCCGGCGCGTTCCGGCGGGCACCGGCGCTCGCG 1160 |
| 9311 | 1121 ACGACAGCCCGGCGCGTTCCGGCGGGCACCGGCGCTCGCG 1160 |
| CSSL128 | 1161 GTCGGCCGAGAGCGTCGGGTTCGACGGCGCCGCGCGGCGG 1200 |
| 9311 | 1161 GTCGGCCGAGAGCGTCGGGTTCGACGGCGCCGCGCGGCGG 1200 |
|  |  |
| CSSL128 | 1201 TCGTCGTCGGCCGCGCCCGTGTCGCCCGACGCGCTCGTCA 1240 |
| 9311 | 1201 TCGTCGTCGGCCGCGCCTGTGTCGCCCGACGCGCTCGTCA 1240 |
|  |  |
| CSSL128 | 1241 GGGTGGGCAGGCTGGTCGACGGCTTCTTGATCGAGGTTGC 1280 |
| 9311 | 1241 GGGTGGGCAGGCTGGTCGACGGCTTCTTGATCGAGGTTGC 1280 |
|  |  |
| CSSL128 | 1281 CAGGGACCCCAACATGCCGCTCGACAAGTTGCTCGCCATT 1320 |
| 9311 | 1281 CAGGGACCCCAACATGCCGCTCGACAAGTTGCTCGCCATT 1320 |
|  |  |
| CSSL128 | 1321 GCCGAGGCCGTCCCGGACACCGCCCGCCCCGAGCACGACG 1360 |
| 9311 | 1321 GCCGAGGCCGTCCCGGACACCGCCCGCCCCGAGCACGACG 1360 |
|  |  |
| CSSL128 | 1361 GCCTCTACAAAGTCGTCGACACTTACCTCAAGGTGCACTC 1400 |
| 9311 | 1361 GCCTCTACAAAGTCGTCGACACTTACCTCAAGGTGCACTC 1400 |

| CSSL128 | 1401 GGAGATGAGCAAGAGCGCGAGGAAGCGGCTGTGCAGGGTG 1440 |
| --- | --- |
| 9311 | 1401 GGAGATGAGCAAGAGCGCGAGGAAGCGGCTGTGCAGGGTG 1440 |
|  |  |
| CSSL128 | 1441 ATCAACTGCAGGAAGCTGTCGGACAAGGCGTGCGCGCACG 1480 |
| 9311 | 1441 ATCAACTGCAGGAAGCTGTCGGACAAGGCGTGCGCGCACG 1480 |
|  |  |
| CSSL128 | 1481 CGGCGCAGAACGAGCTCCTCCCGCTGCGGGTGGTGGTGCA 1520 |
| 9311 | 1481 CGGCGCAGAACGAGCTCCTCCCGCTGCGGGTGGTGGTGCA 1520 |
|  |  |
| CSSL128 | 1521 GGTGCTCTTCTTCGAGCACGCGCGGGCGGCGGCGATGGCG 1560 |
| 9311 | 1521 GGTGCTCTTCTTCGAGCACGCGCGGGCGGCGGCGATGGCG 1560 |
|  |  |
| CSSL128 | 1561 GGCGGCGCGCACGCCGCGGCCGAGCTGCCGGGCAGCATCA 1600 |
| 9311 | 1561 GGCGGCGCGCACGCCGCGGCCGAGCTGCCGGGCAGCATCA 1600 |

| CSSL128 | 1601 GGGCGCTGCTGCAGTCCAAGTCGTCCGGGTCGGATCAGGA 1640 |
| --- | --- |
| 9311 | 1601 GGGCGCTGCTGCAGTCCAAGTCGTCCGGGTCGGATCAGGA 1640 |
|  |  |
| CSSL128 | 1641 GGACGACGCGGCGGACCGCGTGGACGAGCAGCGGCTGCGC 1680 |
| 9311 | 1641 GGACGACGCGGCGGACCGCGTGGACGAGCAGCGGCTGCGC 1680 |
|  |  |
| CSSL128 | 1681 GCGCTCGCCGCAGGCGCGTCCCCCGGGGACGACTGGAGCG 1720 |
| 9311 | 1681 GCGCTCGCCGCCGGCGCGTCCCCCGGGGACGACTGGAGCG 1720 |
|  |  |
| CSSL128 | 1721 TGGAGGGCCTGCGGCGCGCGGCGTCCAAGATCGCCACGCT 1760 |
| 9311 | 1721 TGGAGGGCCTGCGGCGCGCGGCGTCCAAGATCGCCACGCT 1760 |
| CSSL128 | 1761 GCGGATGAAGCTGGAGGAGGACGACGACCACGACGGCGGT 1800 |
| 9311 | 1761 GCGGATGAAGCTGGAGGAGGACGACGACCACGACGGCGGC 1800 |

| CSSL128 | 1801 GGCGGCGACGACGAGGAGTTCGCGCGCAGGCAGCAGGCCG 1840 |
| --- | --- |
| 9311 | 1801 GGCGGCGACGACGAGGAGTTCGCGCGCAGGCAGCAGGCCG 1840 |
|  |  |
| CSSL128 | 1841 GGCTGGCGCGCAGCGCATCGCTGCGGTTCAGGGCGTTCTG 1880 |
| 9311 | 1841 GGCTGGCGCGCAGCGCATCGCTGCGGTTCAGGGCGTTCTG 1880 |
|  |  |
| CSSL128 | 1881 CGCCATCCCGGCGGCGAGGCCGAAGCGGATGCTCAGCAAG 1920 |
| 9311 | 1881 CGCCATCCCGGCGGCGAGGCCGAAGCGGATGCTCAGCAAG 1920 |
|  |  |
| CSSL128 | 1921 CTGTGGCCGCTGGCCAGAGGCGTCACCACCGAGCGGCATT 1960 |
| 9311 | 1921 CTGTGGCCGCTGGCCAGAGGCGTCACCACCGAGCGGCATT 1960 |
|  |  |
| CSSL128 | 1961 AG 1962 |
| 9311 | 1961 AG 1962 |

| CSSL128 | 1 MKFMKLGTRPDTFYSNESVRSVCTEVATDLQILVGDCLYQ 40 |
| --- | --- |
| 9311 | 1 MKFMKLGTRPDTFYSNESVRSVCTEVATDLQILVGDCLYQ 40 |
|  |  |
| CSSL128 | 41 LHKFPLLSKCLLLQALCAESGCGGNGGDVIELPGFPGGVE 80 |
| 9311 | 41 LHKFPLLSKCLLLQALCAESGCGGNGGDVIELPGFPGGVE 80 |
|  |  |
| CSSL128 | 81 AFDACAKFCYGITVTVSARNLVPLRCAAAHLGMSEAADRG 120 |
| 9311 | 81 AFDACAKFCYGITVTVSARNLVPLRCAAAHLGMSEAADRG 120 |
|  |  |
| CSSL128 | 121NLAAKLDAFLASCLLRRWKDALAVLNSTRHCAPLCEDIGL 160 |
| 9311 | 121 NLAAKLDAFLASCLLRRWKDALAVLNSTRHCAPLCEDIGL 160 |
|  |  |
| CSSL128 | 161 TSRCVDAVAALIASPAALPAHSSSASPWWAHDVAELGVDL 200 |
| 9311 | 161 TSRCVDAVAALIASPAALPAHSSSASPWWAHDVAELGVDL 200 |

| CSSL128 | 201 FWRIMVAVKATGAVHEKTVGDALKAYARRWLPNVAKDGIV 240 |
| --- | --- |
| 9311 | 201 FWRIMVAVKATGAVHEKTVGDALKAYARRWLPNVAKDGIV 240 |
|  |  |
| CSSL128 | 241 VGADQPFDGAGNGGDGGNASVKQIATRHRLLLEKIVSLIP 280 |
| 9311 | 241 VGADQPFDGAGNGGDGGNASVKQIATRHRLLLEKIVSLIP 280 |
|  |  |
| CSSL128 | 281 AERDAVSCSFLLKLLKAANILSASATSRAELVRRVAWQLE 320 |
| 9311 | 281 AERDAVSCSFLLKLLKAANILSASATSRAELVRRVAWQLE 320 |
|  |  |
| CSSL128 | 321 EATVGDLLIPSLSCVSETLYDVDAVAAILDEFALRHAAAP 360 |
| 9311 | 321 EATVGDLLIPSLSCVSETLYDVDAVDAILDEFALRHAAAP 360 |
| CSSL128 | 361 PPPVALAVSPDDDDDSPARSGGHRRSRSAESVGFDGAARR 400 |
| 9311 | 361 PPPVALAVSPDDDDDSPARSGGHRRSRSAESVGFDGAARR 400 |

| CSSL128 | 401 SSSAAPVSPDALVRVGRLVDGFLIEVARDPNMPLDKLLAI 440 |
| --- | --- |
| 9311 | 401 SSSAAPVSLDALVRVGRLVDGFLIEVARDPNMPLDKLLAI 440 |
|  |  |
| CSSL128 | 441 AEAVPDTARPEHDGLYKVVDTYLKVHSEMSKSARKRLCRV 480 |
| 9311 | 441 AEAVPDTARPEHDGLYKVVDTYLKVHSEMSKSARKRLCRV 480 |
|  |  |
| CSSL128 | 481 INCRKLSDKACAHAAQNELLPLRVVVQVLFFEHARAAAMA 520 |
| 9311 | 481 INCRKLSDKACAHAAQNELLPLRVVVQVLFFEHARAAAMA 520 |
|  |  |
| CSSL128 | 521 GGAHAAAELPGSIRALLQSKSSGSDQEDDAADRVDEQRLR 560 |
| 9311 | 521 GGAHAAAELPGSIRALLQSKSSGSDQEDDAADRVDEQRLR 560 |
|  |  |
| CSSL128 | 561 ALAAGASPGDDWSVEGLRRAASKIATLRMKLEEDDDHDGG 600 |
| 9311 | 561 ALAAGASPGDDWSVEGLRRAASKIATLRMKLEEDDDHDGG 600 |

| CSSL128 | 601 GGDDEEFARRQQAGLARSASLRFRAFCAIPAARPKRMLSK 640 |
| --- | --- |
| 9311 | 601 GGDDEEFARRQQAGLARSASLRFRAFCAIPAARPKRMLSK 640 |
|  |  |
| CSSL128 | 641 LWPLARGVTTERH* 654 |
| 9311 | 641 LWPLARGVTTERH* 654 |
|  |  |
|  |  |
|  |  |
|  |  |

**Os02g0594800**

| CSSL128 | 1 ATGGCGAGGTCTTGGCTTATAACTGGTAGGGGAGTTGCTA 40 |
| --- | --- |
| 9311 | 1 ATGGCGAGGTCTTGGCTTATAACTGGTAGGGGAGTTGCTA 40 |
|  |  |
| CSSL128 | 41 AGAAAATAAGAAATGCACCTCATTGCTCTAGTCGCCCAAT 80 |
| 9311 | 41 AGAAAATAAGAAATGCACCTCATTGCTCTAGTCGCCCAAT 80 |
|  |  |
| CSSL128 | 81 AAGTGAATTGGGTGCAGAAGCACAGATGGAATGTCCAAAC 120 |
| 9311 | 81 AAGTGAATTGGGTGCAGAAGCACAGATGGAATGTCCAAAC 120 |
|  |  |
| CSSL128 | 121 TGCAAACATGTTATTGATAACAGTGATGTTGCTATACAGT 160 |
| 9311 | 121 TGCAAACATGTTATTGATAACAGTGATGTTGCTATACAGT 160 |
|  |  |
| CSSL128 | 161 GGCCTGGGCTGCCTGCTGGTGTCAAGTTTGATCCATCTGA 200 |
| 9311 | 161 GGCCTGGGCTGCCTGCTGGTGTCAAGTTTGATCCATCTGA 200 |

| CSSL128 | 201 TTTGGAATTGCTTGAACATTTGGAACAAAAGATTGGCCTG 240 |
| --- | --- |
| 9311 | 201 TTTGGAATTGCTTGAACATTTGGAACAAAAGATTGGCCTG 240 |
|  |  |
| CSSL128 | 241 GGGGGTTCAAAGCCACATACATTCATTGATGAATTTATTC 280 |
| 9311 | 241 GGGGGTTCAAAGCCACATACATTCATTGATGAATTTATTC 280 |
|  |  |
| CSSL128 | 281 CGACTATAGATAATGATGAAGGGATATGCTATTCACATCC 320 |
| 9311 | 281 CGACTATAGATAATGATGAAGGGATATGCTATTCACATCC 320 |
|  |  |
| CSSL128 | 321 GGAAAATCTTCCTGGTATGAAGAAAGATGGAACCAGTGGT 360 |
| 9311 | 321 GGAAAATCTTCCTGGTATGAAGAAAGATGGAACCAGTGGT 360 |
|  |  |
| CSSL128 | 361 CATTTCTTCCATAGAGTTTCAAATGCATATGGTTGTGGTC 400 |
| 9311 | 361 CATTTCTTCCATAGAGTTTCAAATGCATATGGTTGTGGTC 400 |

| CSSL128 | 401 AGCGCAAGCGTCGAAAGATCAGTAACTGTGATCATGTTGT 440 |
| --- | --- |
| 9311 | 401 AGCGCAAGCGTCGAAAGATCAGTAACTGTGATCATGTTGT 440 |
|  |  |
| CSSL128 | 441 TTCTGTTGAACATGTGAGATGGCACAAGACAGGGAAATCC 480 |
| 9311 | 441 TTCTGTTGAACATGTGAGATGGCACAAGACAGGGAAATCC 480 |
|  |  |
| CSSL128 | 481 AAAGCCATAGTTGAAAAGGGGGTTACAAAAGGTTGGAAGA 520 |
| 9311 | 481 AAAGCCATAGTTGAAAAGGGGGTTACAAAAGGTTGGAAGA 520 |
|  |  |
| CSSL128 | 521 AAATAATGGTTCTGTACAAGAGTTCACAAAGAGGTGCCAA 560 |
| 9311 | 521 AAATAATGGTTCTGTACAAGAGTTCACAAAGAGGTGCCAA 560 |
|  |  |
|  |  |
| CSSL128 | 561 ACCTGATAAGGCTAACTGGGTGATGCATCAGTACCATCTT 600 |
| 9311 | 561 ACCTGATAAGGCTAACTGGGTGATGCATCAGTACCATCTT 600 |

| CSSL128 | 601 GGGGCAGAAGAAGATGAAAAGGATGGGGAGCTTGTTGTCT 640 |
| --- | --- |
| 9311 | 601 GGGGCAGAAGAAGATGAAAAGGATGGGGAGCTTGTTGTCT 640 |
|  |  |
| CSSL128 | 641 CCAAAATCTCCTACCAGTTGCATGGAAAACAGATAGACAA 680 |
| 9311 | 641 CCAAAATCTCCTACCAGTTGCATGGAAAACAGATAGACAA 680 |
|  |  |
| CSSL128 | 681 GTCTGAAACAGGAAATGCTGATGAAGAATCTGATGCATTT 720 |
| 9311 | 681 GTTAGAAACAGGAAATGCTGATGAAGAATCTGATGCATTT 720 |
|  |  |
| CSSL128 | 721 GCTGCAAGGGTTGGTCCAAAAACTCCAAAGTCAAACACCC 760 |
| 9311 | 721 GCTGCAAGGGTTGGTCCAAAAACTCCAAAGTCAAACACCC 760 |
|  |  |
| CSSL128 | 761 CACAGCCATGTCGCTTAAAAAACAGTCCATGTGAAACAGA 800 |
| 9311 | 761 CACAGCCATGTCGCTTAAAAAACAGTCCATGTGAAACAGA 800 |

| CSSL128 | 801 AAATTACGATCCCATCTTGGAGGACCAGGACGAAGAGGAG 840 |
| --- | --- |
| 9311 | 801 AAATTACGATCCCATCTTGGAGGACCAGGACGAAGAGGAG 840 |
|  |  |
| CSSL128 | 841 TCTAACATACCTATTGTTAGCCTGAAGGATGATGCTGGGA 880 |
| 9311 | 841 TCTAACATACCTATTGTTAGCCTGAAGGATGATGCTGGGA 880 |
|  |  |
| CSSL128 | 881 ATCCTGCGTGGTGCGCTGGAGAAACCCAGGCCGCGAGGGA 920 |
| 9311 | 881 ATCCTGCGTGGTGCGCTGGAGAAACCCAGGCCGCGAGGGA 920 |
|  |  |
| CSSL128 | 921 AGCAGTTCAGGCCTGTCCTAACTTGGATGAATCCCTGCGC 960 |
| 9311 | 921 AGCAGTTCAGGCCTGTCCTAACTTGGATGAATCCCTGCGC 960 |
|  |  |
| CSSL128 | 961 TGTCATGAAGTTCTGGATTCCTTCTATCATGAAACGTTGC 1000 |
| 9311 | 961 TGTCATGAAGTTCTGGATTCCTTCTATCATGAAACGTTGC 1000 |

| CSSL128 | 1001 TTCCTTCTGACCGCCCAATTCTGTCCCAAGGCGGGAATGA 1040 |
| --- | --- |
| 9311 | 1001 TTCCTTCTGACCGCCCAATTCTGTCCCAAGGCGGGAATGA 1040 |
|  |  |
| CSSL128 | 1041 GATTCTTGACAGGAACCTGAATGCGGTGTATGGACTACCT 1080 |
| 9311 | 1041 GATTCTTGACAGGAACCTGAATGCGGTGTATGGACTACCT 1080 |
|  |  |
| CSSL128 | 1081 GATCTCTACAATGTAGACCTTGGAACACCTCCAGATTTTC 1120 |
| 9311 | 1081 GATCTCTACAATGTAGACCTTGGAACACCTCCAGATTTTC 1120 |
|  |  |
| CSSL128 | 1121 AGCTTGCTGATTTGCAGTTTGGATCACAGGAGAGCATTGG 1160 |
| 9311 | 1121 AGCTTGCTGATTTGCAGTTTGGATCACAGGAGAGCATTGG 1160 |
| CSSL128 | 1161 CAACTGGCTGGATAGCATCTAG 1182 |
| 9311 | 1161 CAACTGGCTGGATAGCATCTAG 1182 |

| CSSL128 | 1 MARSWLITGRGVAKKIRNAPHCSSRPISELGAEAQMECPN 40 |
| --- | --- |
| 9311 | 1 MARSWLITGRGVAKKIRNAPHCSSRPISELGAEAQMECPN 40 |
|  |  |
| CSSL128 | 41 CKHVIDNSDVAIQWPGLPAGVKFDPSDLELLEHLEQKIGL 80 |
| 9311 | 41 CKHVIDNSDVAIQWPGLPAGVKFDPSDLELLEHLEQKIGL 80 |
|  |  |
| CSSL128 | 81 GGSKPHTFIDEFIPTIDNDEGICYSHPENLPGMKKDGTSG 120 |
| 9311 | 81 GGSKPHTFIDEFIPTIDNDEGICYSHPENLPGMKKDGTSG 120 |
|  |  |
| CSSL128 | 121 HFFHRVSNAYGCGQRKRRKISNCDHVVSVEHVRWHKTGKS 160 |
| 9311 | 121 HFFHRVSNAYGCGQRKRRKISNCDHVVSVEHVRWHKTGKS 160 |
|  |  |
| CSSL128 | 161 KAIVEKGVTKGWKKIMVLYKSSQRGAKPDKANWVMHQYHL 200 |
| 9311 | 161 KAIVEKGVTKGWKKIMVLYKSSQRGAKPDKANWVMHQYHL 200 |

| CSSL128 | 201 GAEEDEKDGELVVSKISYQLHGKQIDKSETGNADEESDAF 240 |
| --- | --- |
| 9311 | 201 GAEEDEKDGELVVSKISYQLHGKQIDKLETGNADEESDAF 240 |
|  |  |
| CSSL128 | 241 AARVGPKTPKSNTPQPCRLKNSPCETENYDPILEDQDEEE 280 |
| 9311 | 241 AARVGPKTPKSNTPQPCRLKNSPCETENYDPILEDQDEEE 280 |
|  |  |
| CSSL128 | 281 SNIPIVSSKDDAGNPAWCAGETQAAREAVQACPNLDESLR 320 |
| 9311 | 281 SNIPIVSSKDDAGNPAWCAGETQAAREAVQACPNLDESLR 320 |
|  |  |
| CSSL128 | 321 CHEVLDSFYHETLLPSDRPILSQGGNEILDRNLNAVYGLP 360 |
| 9311 | 321 CHEVLDSFYHETLLPSDRPILSQGGNEILDRNLNAVYGLP 360 |
|  |  |
| CSSL128 | 361DLYNVDLGTPPDFQLADLQFGSQESIGNWLDSI* 394 |
| 9311 | 361DLYNVDLGTPPDFQLADLQFGSQESIGNWLDSI* 394 |

**Os02g0594900**

| CSSL128 | 1 ATGCAGAACTGCTTGCTTGATGCTTCGATTTTGGCTATTT 40 |
| --- | --- |
| 9311 | 1 ATGCAGAACTGCTTGCTTGATGCTTCGATTTTGGCTATTT 40 |
|  |  |
| CSSL128 | 41 TATTGCGTTTGGATCGAATAAAAATGGGATTAGGTGAAGT 80 |
| 9311 | 41 TATTGCGTTTGGATCGAATAAAAATGGGATTAGGTGAAGT 80 |
|  |  |
| CSSL128 | 81 TCAAGCTTGGATGCTTCTGGCGCCGGTAGAATCTCGCGAG 120 |
| 9311 | 81 TCAAGCTTGGATGCTTCTGGCGCCGGTAGAATCTCGCGAG 120 |
|  |  |
| CSSL128 | 121 CTGGCCGAATTGCATGGTTCAAAGGCAATTCTGAGCATGT 160 |
| 9311 | 121 CTGGCCGAATTGCATGGTTCAAAGGCAATTCTGAGCATGT 160 |
|  |  |
| CSSL128 | 161 CAAGGTTGAAGTGTGCGCTTCGGGGGTTTGATTTGAGGGC 200 |
| 9311 | 161 CAAGGTTGAAGTGTGCGCTTCGGGGGTTTGATTTGAGGGC 200 |

| CSSL128 | 201 ACTTCTGATCCTCTTGATTGGTGTGCCAGCTCTAATATTC 240 |
| --- | --- |
| 9311 | 201 ACTTCTGATCCTCTTGATTGGTGTGCCAGCTCTAATATTC 240 |
|  |  |
| CSSL128 | 241 ATTATATATGTTCATGGCCAGAAGGTGACTTACTTTCTTC 280 |
| 9311 | 241 ATTATATATGTTCATGGCCAGAAGGTGACTTACTTTCTTC 280 |
|  |  |
| CSSL128 | 281 GACCGATCTGGGAAAAACCGCCAAAGCCCTTCAATGTGCT 320 |
| 9311 | 281 GACCGATCTGGGAAAAACCGCCAAAGCCCTTCAATGTGCT 320 |
|  |  |
| CSSL128 | 321 TCCTCACTACTATCATGAAAATGTCTCGATGGCAAACCTA 360 |
| 9311 | 321 TCCTCACTACTATCATGAAAATGTCTCGATGGCAAACCTA 360 |
|  |  |
| CSSL128 | 361 TGCAAGTTGCATGGATGGAAAGTCAGGGAAACTCCACGCC 400 |
| 9311 | 361 TGCAGGTTGCATGGATGGAAAGTCAGGGAAACTCCACGCC 400 |

| CSSL128 | 401 GTGTTTTTGATGCTGTGCTTTTCAGCAATGAGCTTGACAT 440 |
| --- | --- |
| 9311 | 401 GTGTTTTTGATGCTGTGCTTTTCAGCAATGAGCTTGACAT 440 |
|  |  |
| CSSL128 | 441 TCTTGATATCCGTTGGCATGAGCTTAGCCCATATGTGTCA 480 |
| 9311 | 441 TCTTGATATCCGTTGGCATGAGCTTAGCCCATATGTGTCA 480 |
|  |  |
| CSSL128 | 481 GAATTTGTGCTGCTCGAGTCCAACTCAACCTTCACTGGCC 520 |
| 9311 | 481 GAATTTGTGCTGCTCGAGTCCAACTCAACCTTCACTGGCC 520 |
|  |  |
| CSSL128 | 521 TGAAAAAGGATCTCCACTTCAAGGAAAACCGTCAACGTTT 560 |
| 9311 | 521 TGAAAAAGGATCTCCACTTCAAGGAAAACCGTCAACGTTT 560 |
|  |  |
|  |  |
| CSSL128 | 561 TGAATTTGCTGAATCACGGTTGACCTATGGTATGATAGGT 600 |
| 9311 | 561 TGAATTTGCTGAATCACGGTTGACCTATGGTATGATAGGT 600 |

| CSSL128 | 601 GGAAGATTTGTGAAGGGGGAGAACCCATTTGTCGAGGAGT 640 |
| --- | --- |
| 9311 | 601 GGAAGATTTGTGAAGGGGGAGAACCCATTTGTCGAGGAGT 640 |
|  |  |
| CSSL128 | 641 CATATCAAAGGGTTGCTCTTGACCAGCTTATCAAAATTGC 680 |
| 9311 | 641 CATATCAAAGGGTTGCTCTTGACCAGCTTATCAAAATTGC 680 |
|  |  |
| CSSL128 | 681 TGGAATAACAGATGATGACCTTTTGATCATGTCTGATGTT 720 |
| 9311 | 681 TGGAATCACAGATGATGACCTTTTGATCATGTCTGATGTT 720 |
|  |  |
| CSSL128 | 721 GATGAGATCCCCAGTGGCCATACAATCAACCTCTTGAGGT 760 |
| 9311 | 721 GATGAGATCCCCAGTGGCCATACAATCAACCTCTTGAGGT 760 |
|  |  |
| CSSL128 | 761 GGTGTGATGACACTCCTGAAGTGCTTCATCTCCAGCTCAG 800 |
| 9311 | 761 GGTGTGATGACACTCCTGAAGTGCTTCATCTCCAGCTCAG 800 |

| CSSL128 | 801 GAACTATCTTTACTCATTTCAGTTTCTCCTTGACGACAAG 840 |
| --- | --- |
| 9311 | 801 GAACTATCTTTACTCATTTCAGTTTCTCCTTGACGACAAG 840 |
|  |  |
| CSSL128 | 841 AGTTGGAGGGCTTCAATACACAGATACCGGGCTGGAAAGA 880 |
| 9311 | 841 AGTTGGAGGGCTTCAATACACAGATACCGGGCTGGAAAGA 880 |
|  |  |
| CSSL128 | 881 CGAGGTATGCGCATTTCCGGCAAACAGACGACCTTCTGGC 920 |
| 9311 | 881 CGAGGTATGCGCATTTCCGGCAAACAGACGACCTTCTGGC 920 |
|  |  |
| CSSL128 | 921 TGACTCAGGGTGGCACTGCAGCTTTTGCTTCCGGCACATA 960 |
| 9311 | 921 CGACTCAGGGTGGCACTGCAGCTTTTGCTTCCGGCACATA 960 |
|  |  |
| CSSL128 | 961 AATGATTTTGTCTTCAAAATGCAAGCTTACAGCCATGTTG 1000 |
| 9311 | 961 AATGATTTTGTCTTCAAAATGCAAGCTTACAGCCATGTTG 1000 |

| CSSL128 | 1001 ACCGGATTAGATTTAAGTACTTCTTGAACCCCAAAAGGAT 1040 |
| --- | --- |
| 9311 | 1001 ACCGGATTAGATTTAAGTACTTCTTGAACCCCAAAAGGAT 1040 |
|  |  |
| CSSL128 | 1041 TCAGCATGTGATATGCCAAGGAGCTGATCTTTTTGACATG 1080 |
| 9311 | 1041 TCAGCATGTGATATGCCAAGGAGCTGATCTTTTTGACATG 1080 |
|  |  |
| CSSL128 | 1081 CTTCCCGAAGAGTATACATTCCAAGAGATCATTGCCAAGT 1120 |
| 9311 | 1081 CTTCCCGAAGAGTATACATTCCAAGAGATCATTGCCAAGT 1120 |
|  |  |
| CSSL128 | 1121 TGGGGCCAATCCCTAGCACATTTTCAGCTGTTCACCTTCC 1160 |
| 9311 | 1121 TGGGGCCAATCCCTAGCACATTTTCAGCTGTTCACCTTCC 1160 |
| CSSL128 | 1161 TGCTTATCTGTTGGAGAAAATGGACCAGTACCGCTATCTT 1200 |
| 9311 | 1161 TGCTTATCTGTTGGAGAAAATGGACCAGTACCGCTATCTT 1200 |

| CSSL128 | 1201CTTCCGGGCAACTGCATGCGAGAAAGTGGCTAG 1233 |
| --- | --- |
| 9311 | 1201CTTCCGGGCAACTGCATGCGAGAAAGTGGCTAG 1233 |

| CSSL128 | 1 MQNCLLDASILAILLRLDRIKMGLGEVQAWMLLAPVESRE 40 |
| --- | --- |
| 9311 | 1 MQNCLLDASILAILLRLDRIKMGLGEVQAWMLLAPVESRE 40 |
|  |  |
| CSSL128 | 41 LAELHGSKAILSMSRLKCALRGFDLRALLILLIGVPALIF 80 |
| 9311 | 41 LAELHGSKAILSMSRLKCALRGFDLRALLILLIGVPALIF 80 |
|  |  |
| CSSL128 | 81 IIYVHGQKVTYFLRPIWEKPPKPFNVLPHYYHENVSMANL 120 |
| 9311 | 81 IIYVHGQKVTYFLRPIWEKPPKPFNVLPHYYHENVSMANL 120 |
|  |  |
| CSSL128 | 121 CKLHGWKVRETPRRVFDAVLFSNELDILDIRWHELSPYVS 160 |
| 9311 | 121 CRLHGWKVRETPRRVFDAVLFSNELDILDIRWHELSPYVS 160 |
|  |  |
| CSSL128 | 161 EFVLLESNSTFTGLKKDLHFKENRQRFEFAESRLTYGMIG 200 |
| 9311 | 161 EFVLLESNSTFTGLKKDLHFKENRQRFEFAESRLTYGMIG 200 |

| CSSL128 | 201 GRFVKGENPFVEESYQRVALDQLIKIAGITDDDLLIMSDV 240 |
| --- | --- |
| 9311 | 201 GRFVKGENPFVEESYQRVALDQLIKIAGITDDDLLIMSDV 240 |
|  |  |
| CSSL128 | 241 DEIPSGHTINLLRWCDDTPEVLHLQLRNYLYSFQFLLDDK 280 |
| 9311 | 241 DEIPSGHTINLLRWCDDTPEVLHLQLRNYLYSFQFLLDDK 280 |
|  |  |
| CSSL128 | 281 SWRASIHRYRAGKTRYAHFRQTDDLLADSGWHCSFCFRHI 320 |
| 9311 | 281 SWRASIHRYRAGKTRYAHFRQTDDLLADSGWHCSFCFRHI 320 |
|  |  |
| CSSL128 | 321 NDFVFKMQAYSHVDRIRFKYFLNPKRIQHVICQGADLFDM 360 |
| 9311 | 321 NDFVFKMQAYSHVDRIRFKYFLNPKRIQHVICQGADLFDM 360 |
|  |  |
| CSSL128 | 361 LPEEYTFQEIIAKLGPIPSTFSAVHLPAYLLEKMDQYRYL 400 |
| 9311 | 361 LPEEYTFQEIIAKLGPIPSTFSAVHLPAYLLEKMDQYRYL 400 |

| CSSL128 | 401LPGNCMRESG* 411 |
| --- | --- |
| 9311 | 401LPGNCMRESG* 411 |
|  |  |
|  |  |
|  |  |

**Os02g0595100**

| CSSL128 | 1 ATGGAGGCCGGCGGGTACTACAACTGCAAGAAGACGGACG40 |
| --- | --- |
| 9311 | 1 ATGGAGGCCGGCGGGTACTACAACTGCAAGAAGACGGACG40 |
|  |  |
| CSSL128 | 41 GCATCTGCGAGGACGTCTGCGACAGCGAGCATGGTTCAAA 80 |
| 9311 | 41 GCATCTGCGAGGACGTCTGCGACAGCGAGCATGGTTCAAA 80 |
|  |  |
| CSSL128 | 81 GGCAGTTTTTAGTATGTCAAGGCTGAAGTGCGCACTTCGG120 |
| 9311 | 81 GGCAGTTTTTAGTATGTCAAGGCTGAAGTGCGCACTTCGG120 |
|  |  |
| CSSL128 | 121 GGGTTTGATTTAAGAGCACTTTTGATCCTCTTGATTGGTC 160 |
| 9311 | 121 GGGTTTGATTTAAGAGCACTTTTGATCCTCTTGATTGGTC 160 |
|  |  |
| CSSL128 | 161 TGCCAATTCTGATCTTTGTTATATACCTTCATGGCCAGAA 200 |
| 9311 | 161 TGCCAATTCTGATCTTTGTTATATACCTTCATGGCCAGAA 200 |

| CSSL128 | 201 AGTCACTTACTTCCTCAGACCAATCTGGGAAAAGCCCCCA 240 |
| --- | --- |
| 9311 | 201 AGTCACTTACTTCCTCAGACCAATCTGGGAAAAGCCCCCA 240 |
|  |  |
| CSSL128 | 241 AAGCCCTTCAAAGTACTTCCTCACTACTTCAACGAAAATG 280 |
| 9311 | 241 AAGCCCTTCAAAGTACTTCCTCACTACTACAACGAAAATG 280 |
|  |  |
| CSSL128 | 281 TTTCAATGTCCAACTTATGCAAGTTACATGGATGGAAAGT 320 |
| 9311 | 281 TCTCAATGGCCAACTTATGCAAGTTACATGGATGGAAAGT 320 |
|  |  |
| CSSL128 | 321 CAGGGAAACGCCGCGGCGTGTTTTTGATGCTGTGCTTTTC 360 |
| 9311 | 321 CAGGGAAACGCCGCGGCGGGTTTTTGATGCTGTGCTTTTC 360 |
|  |  |
| CSSL128 | 361 AGCAATGAGCTTGATATTCTTGATATCCGTTGGCATGAGC 400 |
| 9311 | 361 AGCAACGAGCTTGATATTCTTGATATCCGTTGGCATGAGC 400 |

| CSSL128 | 401 TTAGCCCATATGTGTCAGAGTTTGTGTTGCTTGAGTCCAA 440 |
| --- | --- |
| 9311 | 401 TTAGCCCTTATGTGTCAGAGTTTGTGTTGCTTGAGTCCAA 440 |
|  |  |
| CSSL128 | 441 CTCAACCTTCACTGGCCTTAAAAAGGATCTCCACTTCAAG 480 |
| 9311 | 441 CTCAACCTTCACTGGCCTTAAAAAGGATCTCCACTTCAAG 480 |
|  |  |
| CSSL128 | 481 GAAAACCGTCAACGTTTTGAATTTGCTGAATCACGGTTGA 520 |
| 9311 | 481 GAAAACCGTCAACGTTTTGAATTTGCTGAATCACGGTTGA 520 |
|  |  |
| CSSL128 | 521 CCTATGGTATGATAGGTGGTCGGTTTGTGAAGGGTGAGAA 560 |
| 9311 | 521 CCTATGGTATGATAGGTGGTCGGTTTGTGAAGGGGGAGAA 560 |
|  |  |
|  |  |
| CSSL128 | 561 CCCATTTGTCGAGGAGTCATATCAGAGGGTTGCTCTTGAC 600 |
| 9311 | 561 CCCATTTGTCGAGGAGTCATATCAGAGGGTTGCTCTTGAC 600 |

| CSSL128 | 601 CAGCTTATTAAAATTGCCGGGATCACAGATGATGACCTGT 640 |
| --- | --- |
| 9311 | 601 CAGCTTATTAAAATTGCCGGGATCACAGATGATGACCTGT 640 |
|  |  |
| CSSL128 | 641 TGATCATGTCTGATGTTGATGAGATTCCAAGTGGGCATAC 680 |
| 9311 | 641 TGATCATGTCTGATGTTGATGAGATTCCAAGTGGGCATAC 680 |
|  |  |
| CSSL128 | 681 GATCAACCTCTTGAGATGGTGTGATGACATACCTGAAGTA 720 |
| 9311 | 681 GATCAACCTCTTGAGATGGTGTGATGACATACCTGAAGTA 720 |
|  |  |
| CSSL128 | 721 CTCCATCTCCAGCTCAGGAACTATCTGTACTCATTTGAAT 760 |
| 9311 | 721 CTCCATCTCCAGCTCAGGAACTATCTGTACTCATTTGAAT 760 |
|  |  |
| CSSL128 | 761 TTTTCCTTGATGACAAAAGTTGGAGGGCTTCAATTCACAG 800 |
| 9311 | 761 TTTTCCTTGATGACAAAAGTTGGAGGGCTTCAATTCACAG 800 |

| CSSL128 | 801 ATACCGGGCTGGAAAGACGAGGTATGCACATTTCCGGCAA 840 |
| --- | --- |
| 9311 | 801 ATACCGGGCTGGAAAGACGAGATATGCACATTTCCGGCAA 840 |
|  |  |
| CSSL128 | 841 ACAGATGACCTTCTGGCTGATTCGGGATGGCACTGCAGCT 880 |
| 9311 | 841 ACAGATGACCTTCTGGCTGATTCAGGATGGCACTGCAGCT 880 |
|  |  |
| CSSL128 | 881 TTTGCTTTCGCTACATTAGTGATTTTGTCTTCAAAATGCA 920 |
| 9311 | 881 TTTGCTTTCGCTACATTAGTGATTTTGTCTTCAAAATGCA 920 |
|  |  |
| CSSL128 | 921 AGCTTATAGCCATGTTGATCGGATTAGATTTAAGTATTTC 960 |
| 9311 | 921 AGCTTATAGCCATGTTGATCGGATTAGATTTAAGTACTTC 960 |
|  |  |
| CSSL128 | 961 CTGAACCCCAAAAGAATTCAGCATGTGATATGCCAAGGAG 1000 |
| 9311 | 961 CTGAACCCCAAAAGAATTCAGCATGTGATATGCCGAGGAG 1000 |

| CSSL128 | 1001 CCGATCTTTTTGACATGCTTCCTGAAGAATACACATTCCA 1040 |
| --- | --- |
| 9311 | 1001 CCGATCTTTTCGACATGCTTCCTGAAGAATACACATTCCA 1040 |
|  |  |
| CSSL128 | 1041 AGAAATCATTGCCAAGCTGGGTCCAATCCCAAGCACATTT 1080 |
| 9311 | 1041 AGAAATCATTGCCAAGCTGGGTCCAATCCCAAGCACATTT 1080 |
|  |  |
| CSSL128 | 1081 TCTGCCGTTCATCTTCCTGCTTATTTGCTAGAGAAAGTAG 1120 |
| 9311 | 1081 TCTGCCGTTCATCTTCCTGCTTATTTGCTAGAGAAAGTAG 1120 |
|  |  |
| CSSL128 | 1121 ACCAGTACAGTTATCTTCTTCCAGGGCGATGCATGAGAGA 1160 |
| 9311 | 1121 ATCAGTACAGTTATCTTCTTCCAGGGCGATGCATGAGAGA 1160 |
| CSSL128 | 1161 GAGTGGCTAG 1170 |
| 9311 | 1161 GAGTGGCTAG 1170 |

| CSSL128 | 1 MEAGGYYNCKKTDGICEDVCDSEHGSKAVFSMSRLKCALR 40 |
| --- | --- |
| 9311 | 1 MEAGGYYNCKKTDGICEDVCDSEHGSKAVFSMSRLKCALR 40 |
|  |  |
| CSSL128 | 41 GFDLRALLILLIGLPILIFVIYLHGQKVTYFLRPIWEKPP 80 |
| 9311 | 41 GFDLRALLILLIGLPILIFVIYLHGQKVTYFLRPIWEKPP 80 |
|  |  |
| CSSL128 | 81 KPFKVLPHYFNENVSMSNLCKLHGWKVRETPRRVFDAVLF 120 |
| 9311 | 81 KPFKVLPHYYNENVSMANLCKLHGWKVRETPRRVFDAVLF 120 |
|  |  |
| CSSL128 | 121 SNELDILDIRWHELSPYVSEFVLLESNSTFTGLKKDLHFK 160 |
| 9311 | 121 SNELDILDIRWHELSPYVSEFVLLESNSTFTGLKKDLHFK 160 |
|  |  |
| CSSL128 | 161 ENRQRFEFAESRLTYGMIGGRFVKGENPFVEESYQRVALD 200 |
| 9311 | 161 ENRQRFEFAESRLTYGMIGGRFVKGENPFVEESYQRVALD 200 |

| CSSL128 | 201 QLIKIAGITDDDLLIMSDVDEIPSGHTINLLRWCDDIPEV 240 |
| --- | --- |
| 9311 | 201 QLIKIAGITDDDLLIMSDVDEIPSGHTINLLRWCDDIPEV 240 |
|  |  |
| CSSL128 | 241 LHLQLRNYLYSFEFFLDDKSWRASIHRYRAGKTRYAHFRQ 280 |
| 9311 | 241 LHLQLRNYLYSFEFFLDDKSWRASIHRYRAGKTRYAHFRQ 280 |
|  |  |
| CSSL128 | 281 TDDLLADSGWHCSFCFRYISDFVFKMQAYSHVDRIRFKYF 320 |
| 9311 | 281 TDDLLADSGWHCSFCFRYISDFVFKMQAYSHVDRIRFKYF 320 |
|  |  |
| CSSL128 | 321 LNPKRIQHVICQGADLFDMLPEEYTFQEIIAKLGPIPSTF 360 |
| 9311 | 321 LNPKRIQHVICRGADLFDMLPEEYTFQEIIAKLGPIPSTF 360 |
|  |  |
| CSSL128 | 361SAVHLPAYLLEKVDQYSYLLPGRCMRESG*390 |
| 9311 | 361SAVHLPAYLLEKVDQYSYLLPGRCMRESG*390 |

**Supplemental Table. 2** Transcriptome data of genes related to cytokinin, ethylene, GA and four annotated genes between 9311 and CSSL128

| **Gene ID** | **Gene name** | **Function annotation** | **CSSL128/9311** **Fold change (Log 2)** | **Q value** |
| --- | --- | --- | --- | --- |
| Os01g0197700 | *OsCKX2* | cytokinin dehydrogenase precursor | 9.78** | 0.03 |
| Os03g0786400 | *OsDST* | C2H2 zinc finger protein | 0.64 | 0.05 |
| Os04g0442300 | *OsRR1* | A-type response regulator | 0.58 | 0.12 |
| Os02g0557800 | *OsRR2* | A-type response regulator | 0.52 | 0.05 |
| Os02g0830200 | *OsRR3* | A-type response regulator | 0.45 | 0.03 |
| Os01g0952500 | *OsRR4* | A-type response regulator | 0.6 | 0.04 |
| Os04g0524300 | *OsRR5* | A-type response regulator | 0.17* | 0.01 |
| Os04g0673300 | *OsRR6* | A-type response regulator | 1.48 | 0.13 |
| Os07g0449700 | *OsRR7* | A-type response regulator | 0.48 | 0.04 |
| Os08g0376700 | *OsRR8* | A-type response regulator | 1.41 | 0.09 |
| Os11g0143300 | *OsRR9* | A-type response regulator | 0.73 | 0.03 |
| Os12g0139400 | *OsRR10* | A-type response regulator | 0.78 | 0.05 |
| Os02g0631700 | *OsRR11* | A-type response regulator | 0.69 | 0.04 |
| Os02g0594700 | *ORF1* | protein containing the BTBN3 family NPH3 domain | 0.85 | 0.04 |
| Os02g0594800 | *ORF2* | NAM family protein | 0.38* | 0.03 |
| Os02g0594900 | *ORF3* | glycosyl transferase family protein | 1.42 | 0.05 |
| Os02g0595100 | *ORF4* | glycosyl transferase family protein | 1.17 | 0.03 |
| Os09g0451400 | *OsACO1* | 1-aminocyclopropane-1-carboxylate oxidase | 0.68 | 0.13 |
| Os02g0771600 | *OsACO3* | 1-aminocyclopropane-1-carboxylate oxidase | 1.84 | 0.08 |
| Os11g0186900 | *OsACO4* | 1-aminocyclopropane-1-carboxylate oxidase | 1.43 | 0.11 |
| Os05g0149400 | *OsACO5* | 1-aminocyclopropane-1-carboxylate oxidase | 0.59 | 0.07 |
| Os05g0148700 | *OsACO6* | 1-aminocyclopropane-1-carboxylate oxidase | 0.73 | 0.005 |
| Os04g0578000 | *OsACS2* | 1-aminocyclopropane-1-carboxylate oxidase | 0.68 | 0.01 |
| Os06g0130400 | *OsACS6* | aminotransferase | 1.24 | 0.03 |
| Os03g0324300 | *OsEIL1* | ethylene-insensitive3-like 1 protein | 1.37 | 0.14 |
| Os07g0685700 | *OsEIL2* | ethylene-insensitive3-like 1 protein | 0.86 | 0.01 |
| Os07g0155600 | *OsEIN2* | ethylene-insensitive protein | 1.53 | 0.04 |
| Os05g0158600 | *OsGA2ox1* | gibberellin 2-oxidase | 1.73 | 0.11 |
| Os01g0332200 | *OsGA2ox2* | gibberellin oxidase | 0.46 | 0.02 |
| Os01g0757200 | *OsGA2ox3* | gibberellin 2-beta-dioxygenase | 0.42 | 0.23 |
| Os05g0514600 | *OsGA2ox4* | gibberellin 2-beta-dioxygenase | 0.24 | 0.07 |
| Os07g0103500 | *OsGA2ox5* | gibberellin 2-beta-dioxygenase | 5.37** | 0.01 |
| Os05g0560900 | *OsGA2ox8* | gibberellin 2-beta-dioxygenase | 1.84 | 0.09 |
| Os05g0208500 | *OsGA2ox10* | gibberellin 2-beta-dioxygenase | 0.33 | 0.24 |
| Os06g0568600 | *OsKOS2* | ent-kaurene oxidase | 1.81 | 0.15 |
| Os03g0707600 | *OsSLR1* | GRAS family transcription factor domain containing protein | 1.13 | 0.18 |

*Significance at *p*< 0.05; **Significance at *p* <0.01

**Supplemental Table 3** List of primers for molecular mapping and qRT-PCR

| Name | Forward primer (5' to 3') | Reverse primer (5' to 3') |
| --- | --- | --- |
| P1 | AGACGAAAGATCAAACGC | TGGTGTAGGACGAGAAGC |
| P2 | GTCTTTTCTTCTCCAACTT | GCTACACCACCACTTCAT |
| P3 | TTTTGCGATCAGTGTTCGAG | CCTGCGAACTCCTGTTTTTC |
| P4 | GGATGTGAGTGTGGTGTT | CCAAGCAACAGTAGCCAGCA |
| P5 | GGATGTGAGTGTGGTGTT | ATAATGGAAGTATTCAAAATGT |
| P6 | AATAGTTGTTCTCAGTAAGC | GACTACACATATAGCATAAG |
| P7 | TGTGCGATACTGCTCAAGTT | GGAAGTAGGTTGAGAGGTTA |
| *UBI*-QRT | CTGTCAACTGCCGCAAGAAG | GGCGAGTGACGCTCTAGTTC |
| ORF1-QRT | CATGCCGCTCGACAAGTT | GTTGATCACCCTGCACAGC |
| ORF2-QRT | TGGTGGGTGACTGCTTGTAT | CGGGAGCTCGATCACGTCG |
| ORF3-QRT | CAGAAGCACAGATGGAATGT | TTGACACCAGCAGGCAGC |
| ORF4-QRT | AGAATCTCGCGAGCTGGC | AGAGGATCAGAAGTGCCCTC |
| *OsCKX2*-QRT | CGGCAACAAGTGGGACAGTAA | CAGGGCGATGTAGGAAAGC |
| *OsDST*-QRT | ATCCAAGAAGGCAAGGTCAATC | ACACACGAGGAGGAATTGGAA |
| *OsRR1*-QRT | AGGATCAGCAGATGCATGAATG | GAGACGCTGTACGTCCTTGCTT |
| *OsRR2*-QRT | ACGATCTTCTCAAAGCCATCAAG | TGAGAGGCTTAAGGATGAAATCCT |
| *OsRR3*-QRT | GTTACGACGGTGGATAGTGG | ATTCTCCGACGACATTAGCA |
| *OsRR4*-QRT | CTGATCGACAGGAAGCTCAT | CAGGCATGCAGTAGTCAGTG |
| *OsRR5*-QRT | AACCGAATGTGAGCATGATT | AAGCACCTGTTGATCCTTGT |
| *OsRR6*-QRT | GAGCTCCAAGTACAGGGTGA | GAGCTGAGACGATTCCTTGA |
| *OsRR7*-QRT | TGCTCAAGAAGATCAAGGAATCG | GGCACGTTCTCTGACGACATTAT |
| *OsRR8*-QRT | TGTTTTTGGACTCGGAACAT | CCATCCAAGCACTTTCTGAT |
| *OsRR9*/*10*-QRT | ACTGAAAAGCAGATGCAAGG | GCCTTGGTCTTATTGTGTGG |
| *OsRR11*-QRT | AATCACGGACTACTGGATGC | GGCTTCACCAAGAAATCCTC |
| *OsACO1*-QRT  *OsACO3*- QRT  *OsACO4*- QRT  *OsACO5*- QRT  *OsACO6*- QRT  *OsACS2*- QRT  *OsACS6*- QRT  *OsEIL1*- QRT  *OsEIL2*- QRT  *OsEIN2*- QRT  *OsGA2ox1*-QRT  *OsGA2ox2*-QRT  *OsGA2ox3*-QRT  *OsGA2ox4*-QRT  *OsGA2ox5*-QRT  *OsGA2ox8*-QRT  *OsGA2ox10*-QRT  *OsKOS2*-QRT  *OsSLR1*-QRT | GCAGGTACAAGAGCGTGATG  ACCAACGGCAGGTACAAGAG  GGTTTGAGGAGTGGGGGTTC  GAGTACGTGTTCGGCGAGTA  TGTCTCATTTCTAATCCTAGTGGCA  AATGGAGCAAGAACCACCCC  TGGGCAGACATGAGCAAGTT  TGCTCAAGATGATGGAGGTG  GGGCTGATGACCATGTACG  CAGAACTTTGGGGCAAGTAT  CTCGCTTCAGTGCTATTG  GTTCAGCCAGGTGGTAAA  CAATGGGAGGTTCAAGAG  GAACGGGAGGATGAGGAG  AGCACAGAGGATTGCACCAT  CGATTCCTTCTTCGTCAAC  CTCTTTGCGTGATGGTAG  AAGCATTGTCTGTGATAAGC  GATCGTCACCGTGGTAGAGC | TGCCAGGGTTGTAGAAGGAC  CTCCTTGGCCTCGAACTTGT  ACTGCGGGGTTGGATTCTTT  GCAGATTTTGGCGCCTTGA  CTCGCAAAATGGGTAAGCCG  GTCAAACGTTGCCTTGCCTC  TGTCTACCTGGTCCACCGTAGTAT  GGTCGAAGCGGACCTTCT  TCGCGGATGAAGAAATTAGC  TATTGTTGTCCCTTGCTCGAGAGC  CTAACAGGTCCAGGATCT  CTCTCCTAGCAGGTCAAG  CCAAAGTAGATGAAGGAAAC  TCCGAAGTAGATCATTGACAC  CAGCCTGTTGTCTCCAAGCC  AAGTAGATCACGCTCCTG  TTCGTTAGAACCTGTAAGAC  CCTGTTTGTCAGGGTTCTTG  GAGGGAATCGAACATGGTGG |

**Supplemental Fig. 1 Comparison of some yield related traits between 9311 and CSSL128.**

**
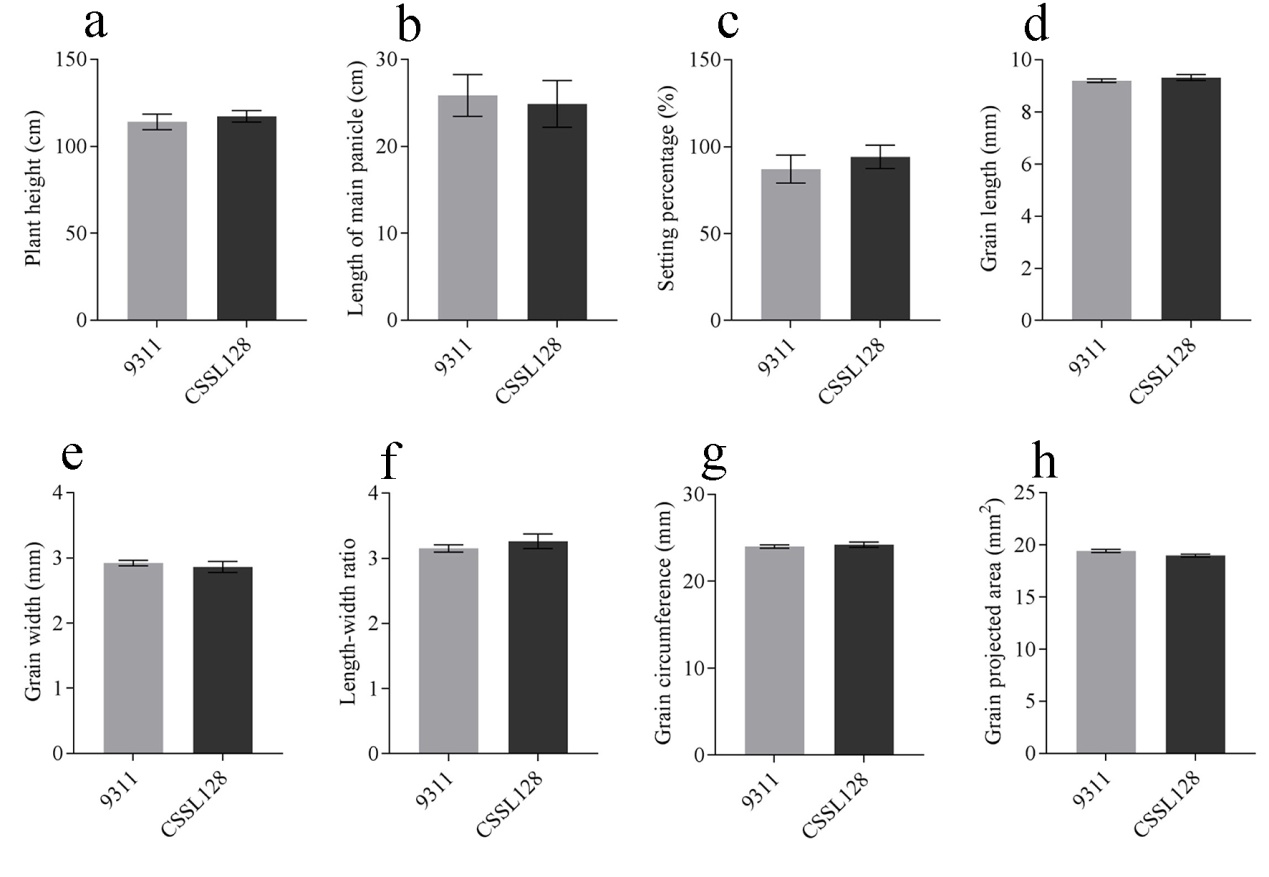
**

**Supplemental Fig. 1** Comparison of plant height (a), length of main panicle (b), setting percentage (c), grain length (d), grain width (e), length-width ratio (f), grain circumference (g) and grain projected area (h) between 9311 and CSSL128. *Significance at *p* < 0.05; **Significance at *p* < 0.01.
